# Gastroprotective and anti-*Helicobacter pylori* potential of herbal formula HZJW: safety and efficacy assessment

**DOI:** 10.1186/1472-6882-13-119

**Published:** 2013-05-30

**Authors:** Jian-Hui Xie, Yun-Long Chen, Qing-He Wu, Jun Wu, Ji-Yan Su, Hong-Yin Cao, Yu-Cui Li, Yi-Sheng Li, Jin-Bin Liao, Xiao-Ping Lai, Ping Huang, Zi-Ren Su

**Affiliations:** 1 School of Chinese Materia Medica, Guangzhou University of Chinese Medicine, Guangzhou 510006, P. R. China; 2 Dongguan Mathematical Engineering Academy of Chinese Medicine, Guangzhou University of Chinese Medicine, Dongguan 523808, P. R. China; 3 Shandong CoIlege of Traditional Chinese Medicine, Yantai 264100, P. R. China; 4 Shenzhen ENT Institute, Shenzhen 518172, P. R. China

**Keywords:** HZJW, TCM, Cytoprotective, Helicobacter pylori, Gastroduodenal ulcer, Safety

## Abstract

**Background:**

A traditional Chinese Medicine (TCM) formula, HZJW, has been applied in clinics in China for gastrointestinal disorders. However, the therapeutic mechanism underlying its efficacy and safety remained to be defined. The present investigation was undertaken to evaluate the formula HZJW for its gastroprotective potential, possible effect on *Helicobacter pylori* along with safety to justify its anti-ulcer action and safe clinical application.

**Methods:**

The gastroduodenal cytoprotective potential was evaluated in rodent experimental models (HCl/Ethanol and NSAID-induced ulcer protocols). The anti-*H*. *pylori* property was assessed by agar dilution assay *in vitro* and analysis *in vivo* including rapid urease test, immunogold test and histopathology. For toxicity assessment, acute toxicity study was performed according to fixed dose procedure with a single oral administration of HZJW to mice. In the oral chronic toxicity, rats (80 males, 80 females) were administrated HZJW orally in 0, 1000, 2500, or 5000 mg/kg/day doses for 26 weeks (*n* = 40/group of each sex). Clinical signs, mortality, body weights, feed consumption, ophthalmology, hematology, serum biochemistry, gross findings, organ weights and histopathology were examined at the end of the 13- and 26-week dosing period, as well as after the 4-week recovery period.

**Results:**

In the HCl/Ethanol-induced ulcer model, it was observed that oral administration with HZJW (260, 520 and 1040 mg/kg) and ranitidine (250 mg/kg) significantly reduced the ulcerative lesion index (116.70 ± 36.4, 102.20 ± 18.20, 84.10 ± 12.1 and 73.70 ± 16.70) in a dose-dependent manner, respectively, with respect to control group (134.10 ± 31.69). Significant inhibition was also observed in ulcerative index from aspirin-induced ulcer model, with decreases of 35.40 ± 5.93, 31.30 ± 8.08, 26.80 ± 8.27and 20.40 ± 6.93 for the groups treated with HZJW and ranitidine, in parallel to controls (41.60 ± 10.80). On the other hand, treatment with HZJW efficaciously eradicated *H*. *pylori* in infected mice in rapid urease test (RUT) and immunogold antibody assay, as further confirmed by reduction of *H*. *pylori* presence in histopathological analysis. In the *in vitro* assay, MICs for HZJW and amoxicillin (positive control) were 125 and 0.12 μg/mL respectively. The LD_50_ of HZJW was over 18.0 g/kg for mice. No drug-induced abnormalities were found as clinical signs, body weight, food consumption, hematology, blood biochemistry, ophthalmology and histopathology results across three doses. No target organ was identified. The No Observed Adverse Effect Level (NOAEL) of HZJW was determined to be 5,000 mg/kg/day for both sexes, a dose that was equivalent to 50 times of human dose.

**Conclusions:**

These results suggested the efficacy and safety of HZJW in healing peptic ulcer and combating *H*. *pylori*, which corroborated their conventional indications and contributed to their antiulcer pharmacological validation, lending more credence to its clinical application for the traditional treatment of stomach complaints symptomatic of peptic ulcer disease (PUD). HZJW might have the potential for further development as a safe and effective alternative/complementary to conventional medication in treating gastrointestinal (GI) disorders.

## Background

Gastric and duodenal ulcers (peptic ulcers) are the most common gastrointestinal disorder that occur in clinical practice and are currently considered as a progressive global health problem. The ulcers that affect the gastrointestinal system are normally provoked by an imbalance between aggressive and protective factors in the stomach [1]. Furthermore, it is also widely accepted that most peptic ulcers are associated with *Helicobacter pylori* infection and eradiation of this organism leads to enhanced ulcer healing and much less chance of ulcer recurrence [2,3].The current medicinal treatment of peptic ulcer is generally based on triple therapy regimen, inhibition of gastric acid secretion by histamine H_2_-antagonists, proton pump inhibitors, as well as on mucosal protective therapy provided by sucralfate and bismuth [4]. However, the treatment is complicated and of high cost, requiring a minimum of two antibiotics in combination with gastric acid inhibitors, which often causes nausea, antibiotic resistance, recurrence and other adverse effects [5-7]. In view of their various harmful adverse effects and the prevalence of antibiotic-resistant *H*. *pylori* strains, the search for safe and effective non-antibiotic agents is essential. In recent years, active researches have rekindled interest in natural drugs possessing these activities, and there has been an increased inclination towards herbal formulations, which are widely appreciated by the population especially in oriental countries.

For centuries, herbals have been used traditionally for the treatment of a wide range of ailments, including gastrointestinal (GI) disorders [8,9], such as dyspepsia, gastritis and peptic ulcer disease (PUD). In China, a variety of Chinese medicinal herbs have been prescribed to attenuate or eradicate gastritis-like disorders and achieved good effects. HZJW, composed of 12 medicinal herbs, is a Chinese herbal formula based on a famous proved recipe proposed by Prof. Honglin Xing (Table 1). HZJW had clinical efficacy of reinforcing spleen and stomach to clear away heat and eliminate dampness, promoting *qi* and blood circulation to relieve pain and flatulence, and had been demonstrated to possess pronounced effectiveness and safety for the treatment of gastrointestinal disease in clinical practice [10,11]. Traditionally, these twelve herbs with well-established histories of application are commonly prescribed together with other herbs in herbal formulas to treat gastrointestinal disorders by Chinese medicine practitioners. According to the theory of TCM, a reasonable combination of medicinal herbs was used to enhance the desired action and eliminate possible side effects. *Corydalis Rhizoma*, *Coptidis Rhizoma* and *Alpinia officinarum*, important components of HZJW, were proved to be effective in the prevention of *H*. *pylori* infection [12,13]. Berberine and its protoberberine alkaloids palmatine, coptisine and aporphinoid alkaloid of magnoflorine have been confirmed to be the major pharmacologically active constituents of *Coptidis Rhizoma* against *H*. *pylori*[14-16]. Berberin, one of the chemical marker of this formula, was also found to possess simultaneous gastrointestine protective and anti-inflammatory activities [15].

**Table 1:** Composition of HZJW

**Linnean classification**	**Botanical origin**	**Weight ratio**
*Macrocephalae Rhizoma*	*Atractylodes macrocephala* Koidz.	0.2
*Scutellariae Barabtae Herba*	*Scutellaria barbata* D. Don	0.15
*Corydalis Rhizoma*	*Corydalis yanhusuo* W.T. Wang	0.12
*Curcumae Radix*	*Curcuma wenyujin* Y.H. Chen et C.Ling	0.12
*Cynanchi Paniculati Radix et Rhizoma*	*Cynanchum paniculatunm* (Bge.) Kitag.	0.12
*Alpiniae Officmarum Rhizoma*	*Alpinia officinarum* Hance	0.03
*Coptidis Rhizoma*	*Coptis chinensis* Franch.	0.06
*Perillae Fructus*	*Perilla frutescens* (L.) Britt.	0.12
*Taraxact Herba*	*Taraxacum mongolicum* Hand. Maza.	0.15
*Typhae Pollen*	*Typha angustifolia* L.	0.12
*Hedyotis diffusa Willd*	*Olydenlandia diffusa* (Wild.) Roxb.	0.3
*Herba Eupatorii*	*Eupatorium fortunei* Turcz.	0.15

In clinical practice in China, we have successfully applied this recipe for treating patients with gastrointestinal disorders. Nevertheless, the evidence-based mechanism of the reputed efficacy remains elusive. No standard preclinical toxicity data in any animal species are currently available to assess its potential toxic effects. The present research was to explore possible mechanisms underlying the pharmacological action of HZJW and unravel its comprehensive toxicological properties. In view of the critical role of cytoprotective and anti-*H*. *pylori* activity in the management of peptic ulcer, the development of a single preparation endowed with both properties will be a welcome contribution. Firstly, we assessed the gastroprotective activity via different rodent experimental models (HCl/Ethanol and NSAID-induced ulcer protocols). Secondly, we determined the anti-*H*. *pylori* effect of HZJW *in vitro* by agar dilution assay and *in vivo* via rapid urease test, immunogold assay and histopathological examination. Thirdly, we undertook an acute toxicity appraisal in Kun-ming mice and a 26-week chronic toxicological study on Sprague–Dawley rats to characterize its possible toxicity. The present scheme should contribute to an improved understanding of possible mechanisms of HZJW and, also valuable information of toxicity to direct its clinical application.

## Methods

### Sources and authentication of herbs

All the herbs of HZJW formula were obtained from Yifang Chinese Material Medica Business Department, Yulin City, all of which are authenticated by Prof. Lai Xiaoping at Guangzhou University of Chinese Medicine. The authenticated voucher specimens (Voucher #20090924) were kept in School of Chinese Materia Medica, Guangzhou University of Chinese Medicine (GUCM). Assurance of quality control for all the materials was validated according to *Pharmacopoeia of the People*'*s Republic of China*[17].

### Preparation of HZJW

HZJW was composed of 12 medicinal herbs as shown in Table 1. *Coptidis Rhizoma* and *Corydalis Rhizoma* were ground to obtain fine powder while others ground to be coarse powder. *Cynanchi Paniculati Radix et Rhizoma* was distilled with water (1:8, w/v) for 4 h and 30% NaCl was added into the obtained distillate to yield salt-outings. After filtration, the residues of *Cynanchi Paniculati Radix et Rhizoma* and the other nine medicinal herbs (except *Coptidis Rhizoma* and *Corydalis Rhizoma*) were extracted with water (1:10, w/v) twice, 1 h each, of which *Scutellariae Barabtae Herba* was added post boiling. The resulting supernatant was concentrated to a relative density (RD) of 1.20 (60°C), mixed together with the abovementioned fine powder thoroughly, and then dried out under vacuum. Finally, the above dry extract was blended thoroughly with the salt-outings and an appropriate amount of microcrystalline cellulose to produce HZJW. The yield of HZJW extract was 31.75% (w/w) compared with the original herbs. Chemical profile of HZJW was analyzed by HPLC (see Additional file 1: Figure S1). A voucher specimen was deposited at GUCM, with the registration number 20091102. HZJW was stored at 4°C and diluted to the desired concentrations in distilled water at the time of administration.

### Bacterial strains and cultivation

*Helicobacter pylori* strain ATCC 43504 (*Vac A* and *Cag A* positives) employed for the assay was obtained from American Type Culture Collection (Rockville, MD), and stored at −80°C in Muller–Hinton broth (OXOID) containing 15% (w/v) glycerol until experimentation. Frozen *H*. *pylori* isolate was thawed and grown on Brucella agar supplemented with bovine serum albumin (BSA) for 72 h at 37°C under a microaerophilic atmosphere (85% N_2_, 10% CO_2_, 5% O_2_) and 98% humidity. Each plate was swabbed with a sterile cotton-tipped applicator, and the tested organism inocula used for the dilution tests were prepared by suspending 72 h colonies in 2 mL of sterile distilled water (DW) to obtain turbidity equivalent to a 2.0 McFarland standard (~10^8^ CFU/mL).

### Experimental animals and maintenance

Sprague–Dawley (SD) rats (180 ± 20 g), Balb/c mice (18 ± 2 g), Kun-ming (KM) mice (18 ± 2 g) of both sexes at the initiation of treatment were obtained from the Medical Experimental Animal Center of Guangzhou University of Chinese Medicine and Evaluation and Research Center for Toxicology Institute of Disease Control and Prevention PLA. Licences for rats and mice were SCXK (YUE) 2008–0020, 2009–0210 and SCXK(JUN)2007-004, respectively. For the experiment, the animals were transferred for the laboratory and submitted to adaptation by period of 7 days. They were acclimatized under controlled temperature (25 ± 2°C) and humidity (50-70%) on a 12-h light/12-h dark cycle (artificial lighting from 08:00 to 20:00) and had free access to standard chow and drinking water. In all experiments, the animals were kept in cages with raised, wide-mesh floors to prevent coprophagy. The experimental protocols involved were in accordance to the rules and guidelines of the Experimental Animal Center of Guangzhou University of Chinese Medicine and, approved by the Animal Care and Use Ethics Committee of our institution, with reference to European Community guidelines for the use of experimental animals.

### Chemicals and drugs

Aspirin was obtained from Bayer HealthCare AG (Lot. BTA8RR3), dexamethasone was obtained from Zhejiang Xianju pharmaceutical Co., LTD (Lot. 091032);ranitidine was purchased from O tevez (foshan) pharmaceutical Co., LTD (Lot. 0908521); amoxicillin was from Zhuhai federal pharmaceutical Co., LTD (Lot. 00800208). Azithromycin was purchased from Suzhou Changzheng Hinkay pharmaceutical Co., LTD (Lot. 100604); gentamycin was obtained from Henan topfond pharmaceutical Co., LTD (Lot. 100630950); ampicillin was obtained from Shandong Lukang pharmaceutical Co., LTD (Lot. L100708). Formaldehyde and phenol red were from Guangzhou Chemical Reagent Factory (Lot. 20100302 & 20091021). Hydrochloric acid, sodium hydroxide and alcohol were purchased from Guangzhou chemical reagent factory (Lot. 20091014, 20090401 & 20090304). *H*. *pylori* urease Immunogold Testing kit was from Beijing Tian Hong Sig biotechnology Co., Ltd (Lot. 010652); *H*. *pylori* infection test paper (Rapid Urease Test, RUT) was from Zhuhai Kedi science and technology development Co., Ltd (Lot. 110401). Scutellarin (Lot. 110842–200605), berberine hydrochloride (Lot. 110713–200910) and paeonol (Lot. 110708–200505) were purchased from National Institute of Food and Drug Control (Beijing, China) with purity over 98% based on HPLC analysis. HPLC-grade ethanol and acetonitril were obtained from Honeywell (Honeywell, USA). All reagents were at least of analytic grade and applied according to the specific instruction manual.

### HCl/Ethanol-induced ulcerogenesis

The experiment was carried out according to the method of Morimoto et al. [18], with the following modifications. Rats of each sex were randomly divided into five groups of ten animals each. The first group was given 1 mL of vehicle (normal saline), and the second group was treated with ranitidine (250 mg/kg, *p*.*o*.). The remaining groups received 260, 520 and 1040 mg/kg (*p*.*o*.) of HZJW respectively. All the treatments were administered daily for 3 consecutive days. All rats were subjected to abrosia 24 h prior to the ulcerogenic challenge. On the 4^th^ day, one hour after the last administration, all rats received an oral dose of 1 mL of 0.15 M HCl in 60% ethanol to induce gastric ulcer. One hour post ulcerogenic challenge, all animals were sacrificed by cervical dislocation, and stomach of each rat was removed and inflated with 10 mL of 1% buffered formalin solution to fix for 10 min. Subsequently, each stomach was incised along the greater curvature and rinsed with normal saline to remove stomach contents, then the lengths of the necrotizing lesions were measured under a dissecting microscope, to access the formation of ulcers (hemorrhagic lesions).The maximum length of each lesion was determined and the sum of lengths of all lesions (mm) for each stomach was expressed as the ulcer index (UI), and the inhibition percentage was calculated by the following formula:

$$\mathrm{Inhibition}\;\left(\%\right)=\left[\left(\text{U}{\text{I}}_{\mathrm{control}}-\text{U}{\text{I}}_{\mathrm{treat}}\right)/\text{U}{\text{I}}_{\mathrm{control}}\right]\times 100\%$$

### Nonsteroidal anti-inflammatory drug (NSAID)-induced ulcer

The experiment was carried out according to the method by Nwafor et al. [19] with a few modifications. Rats of either sex were randomly divided into five groups of ten animals each. The first group was given 1 mL of vehicle (normal saline), and the second group was treated with ranitidine (250 mg/kg, *p*.*o*.). The remaining groups received 260, 520 and 1040 mg/kg (*p*.*o*.) of HZJW respectively. All the treatments were administered daily for 3 consecutive days. All rats were subjected to fast 24 h before the ulcerogenic challenge. On the 4^th^ day, one hour after the last administration, all the rats received an oral dose of aspirin (100 mg/kg) to induce gastric ulcer. Seven hours after the aspirin challenge, all animals were sacrificed by cervical dislocation, and stomach of each rat was excised and inflated with 10 mL of 1% buffered formalin solution to fix for 10 min. Thereafter, the greater curvature of each stomach was incised, and the extent of gastric damage in the glandular region was evaluated according to the ulcerative lesion index and inhibition percentage as described above.

#### *In vitro* anti-*H*. *pylori* assay

Agar dilution test was employed to analyze the susceptibility of reference strain *H*. *pylori* to HZJW in two-fold serial dilution in the range of 500–0.5 mg/mL. *H*. *pylori* were cultivated for 72 hours on blood agar, harvested and suspended in Brucella broth at a final concentration of 2.48 × 10^9^ CFU/mL. Thereafter, a volume of 100 μL bacterial suspension was inoculated in the Brucella medium containing HZJW of serial decreasing concentrations (500–0.5 mg/mL). In addition to the tested agent, amoxicillin was employed as the positive control. Under the same condition, surfaces that were inoculated with a suspension of *H*. *pylori* without any tested materials and non-inoculated media were used as controls. Inocula were incubated at 37 °C under a microaerophilic atmosphere for 72 hours. Minimum inhibitory concentration (MIC), the lowest concentration that inhibits the visible growth of bacterium was determined. This experiment was performed in triplicate.

#### *In vivo* anti-*H*. *pylori* assay

Balb/c mice of either sex (except the normal group that was given normal saline) were pretreated with an oral dose of 0.5 mL antibiotic mixture (ampicillin 10 mg/mL, gentamicin 1.2 mg/mL, azithromycin 10 mg/mL) for 3 consective days, to make sure that they were free from any *H*. *pylori*-like organisms that could have been acquired through natural infection. On the 4th day, mice were intragastricly administrated with 0.5 mL freshly prepared suspension of *H*. *pylori* (10^8^ CFU/mL) daily for 7 days. During this interval, mice were deprived of chew supplement but free access to drinking water was maintained for 12 h each day. On the second day following the last inoculation, the infected animals were randomly grouped according to the following doses: 520, 1040 and 2080 mg/kg HZJW, and were treated for 8 successive weeks (one administration per day), while the control group was given 20 mg/kg normal saline. Amoxicillin, suspended in 0.5% w/v CMC, was used as reference drug, and was administered orally to the mice for 8 successive weeks at a dose of 670 mg/kg body weight, which was approximately 10 times of the maximum recommended human dose. After the last administration, all mice were fasted for 24 h, blood samples were collected from retro-orbital and sacrificed by cervical dislocation. Serum was prepared and subjected to *H*. *pylori* urease Immunogold Testing kit to assess *H*. *pylori* clearance. All procedures were performed as described according to the manufacturers’ recommendation in the kit manuals.

At the same time, the stomach was excised, and the gastric mucosa tissue was submitted to rapid urease test (RUT). In brief, with the aid of tweezers, a fragment of gastric tissue was inserted in the centre of a minitube containing urease gel. Inoculation times were recorded. The minitubes were kept at room temperature and the colour change was evaluated within 3 min. Test result was considered strong positive if an alkaline reaction had developed (from yellow to dark pink within the tissue margin) in 1 min, and weak positive if in 3min, while negative if the colour failed to exhibit any colour variation within 3 min in the medium (yellow or light orange). The rest of the stomach tissues were fixed with a 10% formalin solution, dehydrated, embedded in paraffin, sectioned, deparaffinized, and stained with carbolic acid and basic fuchsin for *H*. *pylori* detection.

### Acute toxicity test of HZJW

Acute oral toxicity study was performed according to fixed dose procedure. Two experimental groups of mice (10 mice of each sex in each group) were treated orally with a single dose of 0 and 18.0 g/kg body weight. Animals receiving the vehicle (saline) served as control. Animals were observed individually at least once during the first 30 min after administration, periodically during the first 24 h (with special attention during the first 4 h) and daily thereafter for a period of 14 days. The observation principally included changes in skin and fur, eyes and mucous membrane (nasal), autonomic changes (salivation, lacrimation, perspiration, piloerection, urinary volume, and defecation) and alterations of the central nervous system (ptosis, drowsiness, gait, tremors and convulsion). Food and water were provided throughout the experiment. For 14 days the animals were weighed and the number of deaths noted.

### Experimental schedule for general toxicity of HZJW

To generally detect potential long-term, repeat-dose toxicity of HZJW and thereby define the characteristic, extent, dose and time-dependent relationship, as well as target organs and tissues of potentially toxic effects, 80 male and 80 female SD rats were assigned randomly and evenly to 4 experimental groups. The animals were separated by gender and housed five in each cage. Group I animals (control) were orally administered with distilled water throughout the course of the study. Animals in Groups II (1000 mg/kg body weight/day), III (2500 mg/ kg body weight/day), IV (5000 mg/kg body weight/day) received orally administered HZJW dissolved in distilled water once-daily for a period of 13 or 26 weeks. The dosages selected in the present study were based on existing data on the effective dose, results of acute toxicity study of HZJW in mice, and suggested human dose (equivalent to 10, 25, and 50 times of the normal human dose in clinical prescription, respectively). Clinical signs (general behavior, fur condition, breathing and nose conditions, eyes and oral secretions, urine and faeces), toxic reactions, and mortality were monitored daily after the initiation of HZJW treatment. Body weight, food and water consumption were recorded once a week. After 13 weeks, 5 rats per each sex in each group were sacrificed. Another 10 rats per each sex in each group were sacrificed at the end of the 26th week. The remaining rats were sacrificed after 4 weeks of recovery. The animals of the recovery groups were observed for reversibility, persistence and delayed occurrence of toxic effects. All examinations were conducted as described above.

At the end of the tested period, all animals were sacrificed and subjected to hematology and clinical chemistry assays, ophthalmic testing (cornea, conjunctiva, iris, pupil, atria, len, fundus, eyelid), necropsy examination, organ weighing, and histopathologic examination. This toxicity study was carried out in compliance with the Testing Guidelines for Safety Evaluation of Drugs (Notification [Z] GPT3-1 issued by China Food and Drug Administration (SFDA) on March 2005) and Good Laboratory Practice Regulations for Nonclinical Laboratory Studies.

### Clinical observation and feed consumption

The animals were observed daily prior to and following administration for signs of toxicity and mortality throughout the experimental period. Detailed clinical signs were assessed and recorded, including changes in skin and fur, eyes and mucous membranes, manure, psyche states and behavior patterns, etc. The body weight was measured at the initiation of treatment and once a week during the treatment period thereafter. The amounts of feed were weighed before they were supplied to each cage and their remnants were measured the next day. The differences were calculated and regarded as daily feed consumption (g/animal/day).

### Haematological and biochemical parameters

Samples of blood were obtained from all animals on Week 13 and Week 26 of the investigation, and after recovery. All rats were fasting but allowed access to water *ad libitum* for more than 12 h prior to blood sample collecting. Blood samples were collected into two tubes: (1) heparinized centrifuge tubes and (2) dry non-heparinized centrifuge tubes. The heparinized blood was used for a hematological determination, which include red blood cell (RBC), hemoglobin (HGB), hematocrit (HCT), mean corpuscular volume (MCV), mean corpuscular HGB (MCH), mean corpuscular HGB concentration (MCHC), red cell distribution width (RDW), platelets (PLT), mean platelet volume (MPV), platelet distribution width (PDW), white blood cell (WBC) counts, reticulocytes (RET), neutrophils (NE), lymphocytes (LYM), monocytes (MON), eosinophils (EOS), and basophils (BAS) with a MEK-7222K Hematology analyzer (Nihon Kohden, Japan) according to the manufacturer’s operator manual. Plasma was isolated and used to determine the prothrombin time (PT) using a coagulometer (CA-50; Sysmex, Japan).

The non-heparinized blood was allowed to coagulate before being centrifuged and the serum separated. Serum was analyzed for changes in biochemistry using an automatic biochemical analyzer (RA-1000, Technico, USA), which measured aspartate aminotransferase activity (AST), alanine aminotransferase activity (ALT), alkaline phosphatase activity (ALP), albumin (ALB), total protein (TP), total cholesterol (CHOL), creatine phosphokinase activity (CPK), total bilirubin (T-BIL), direct bilirubin (D-BIL), creatinine (Cre), triglycerides (TG), urea (Ure) and glucose (GLU). The concentrations of sodium ions (Na^+^), potassium ions (K^+^) and chloride ions (Cl^-^) were measured with an electrolyte autoanalyzer (EasyLyte Na^+^/K^+^/Cl^-^ Analyzer, MEDICA, USA). All parameters of blood chemistry and hematology were measured following standard procedures.

### Organ weights, gross necropsy and histopathological examination

All animals were fasted overnight before scheduled necropsies. A complete necropsy was conducted on all animals and consisted of an external examination, including the identification of all clinically recorded lesions, and a detailed internal examination. The rats were anesthetized with diethyl ether and sacrificed by decapitation after blood collection from the abdominal aorta. After dissection to remove fat and connective tissue, the following organs were carefully dissected out and weighed: brain (cerebrum, cerecellum and brainstem), thymus glands, heart, lungs, liver, spleen, kidneys, adrenal glands, testis/ovary and epididymis/uterus. The relative organ weight was calculated based on the terminal body weight before fixation.

The following organs of all animals were fixed in 10% neutral formalin: brain, thoracic spinal cord, thyroid glands, Gley's glands, pituitary gland, thymus, adrenal glands, esophagus, salivary glands, stomach, small intestine (duodenum, jejunum and ileum), large intestine (cecum, colon and rectum), pancreas, spleen, lungs, trachea, kidneys, liver, heart, aorta, bladder, testes, epididymides, ovaries, uterus, prostate gland, vagina, mammary gland, sciatic nerve, urinary mesenteric lymph node, sternum, sub-mandibular lymph nodes, bone marrow.

The fixed organs of the vehicle control and high dose group and any organs from the other groups that displayed gross abnormalities were subjected to histopathological examination. These samples were Paraffin-embedded, microsectioned at a nominal thickness of 4 μm and then stained with hematoxylin and eosin for histopathological examination.

### Statistical analysis

The data obtained by the various parameters was statistically evaluated by one way analysis of variance (ANOVA), and presented as means ± standard deviation (S.D.) for the indicated number of independently performed experiments. The body and organ weight, food and water consumption, hematological parameters, and blood biochemical parameters were analyzed for homogeneity of dispersion by ANOVA. The parameters found to be significant in ANOVA were assessed by the Dunnett test. Significant differences (as shown in the plots) were classified as: * for *P* < 0.05; more significant ** for *P* < 0.01.

## Results

### Effect of HZJW on HCl/Ethanol-induced gastric ulcer

**Figure 1:**
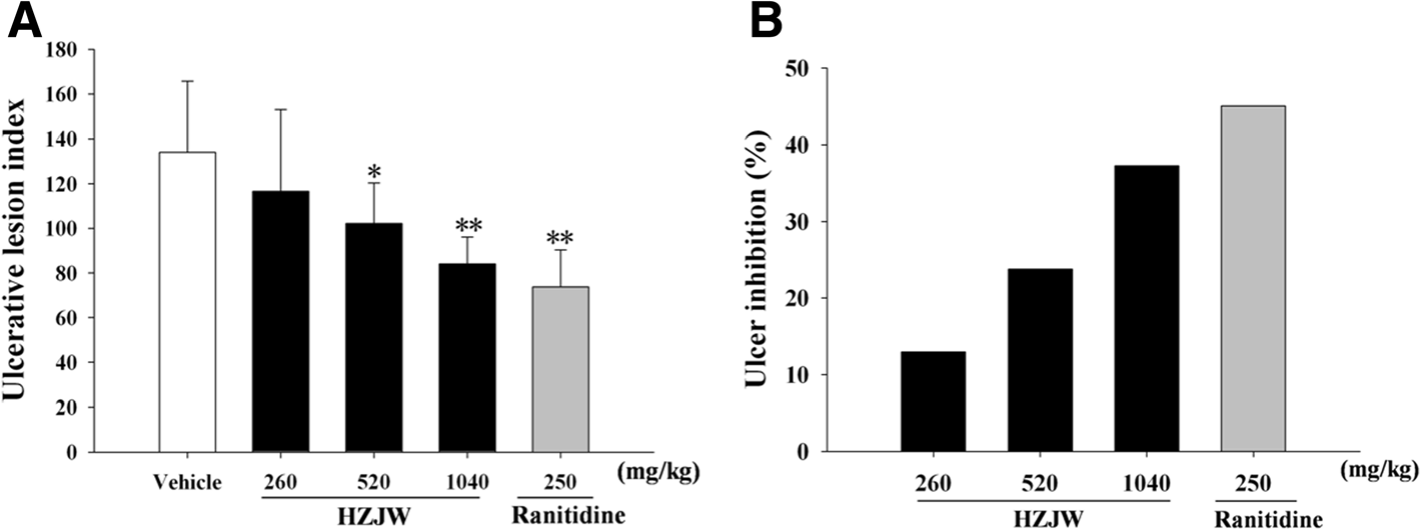
**Protective effect of HZJW and raniditine on ulcerative lesion induced by HCl/Ethanol.** (**A**) Ulcerative lesion index; (**B**) Ulcer inhibition (%). Vehicle group (Open column), ranitidine group (250 mg/kg/d, grey bar), HZJW groups (260, 520, and 1040 mg/kg/d, dark bars). Vertical bars represent standard deviation (S.D.), where *n* = 10. Asterisks designate significant differences: *P < 0.05 & **P < 0.01 vs. Vehicle group.

Ulcerative indices and gastroprotection percentage were determined in rats with HCl/Ethanol-induced ulcers by measuring ulcerative lesion length. As shown in Figure 1, intragastric administration of HCl/Ethanol to rats caused severe gastric mucosal damage (in the form of hemorrhagic streaks), while administration of HZJW displayed statistically significant anti-ulcerogenic activity, and dose-dependently reduced the ulcerative lesion index (116.70 ± 36.4; 102.20 ± 18.20, *P* < 0.05; 84.10 ± 12.1, *P* < 0.01 at doses of 260, 520 and 1040 mg/kg, respectively), as compared with the model group (134.10 ± 31.69). Ranitidine also offered significant protection against HCl/Ethanol-induced gastric lesions (20.40 ± 6.93, *P* < 0.01). The percentages of ulcer inhibition were 12.98%, 23.79%, 37.29% and 45.04% for the groups treated with 260, 520 and 1040 mg/kg of HZJW and ranitidine, respectively.

### Effect of HZJW on NSAID-induced ulcer

**Figure 2:**
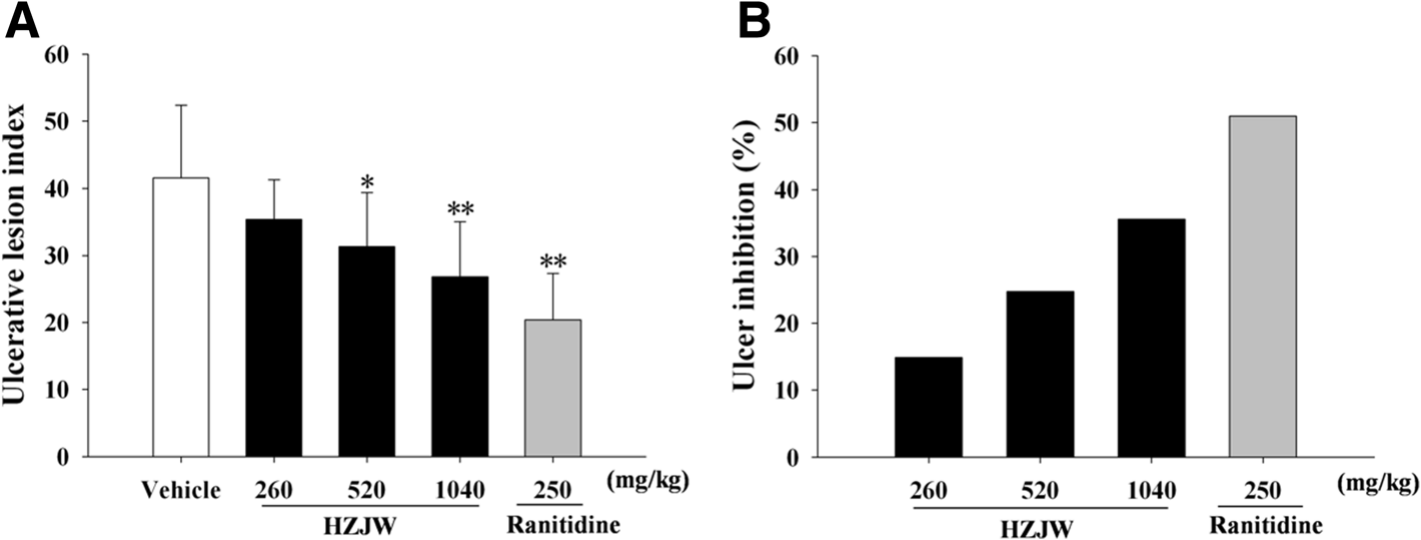
**Protective effect of HZJW and raniditine on ulcerative lesion induced by NSAID.** (**A**) Ulcerative lesion index; (**B**) Ulcer inhibition (%). Vehicle group (Open column), ranitidine group (250 mg/kg/d, grey bar), HZJW groups (260, 520, and 1040 mg/kg/d, dark bars). Vertical bars represent standard deviation (S.D.), where *n* = 10. Asterisks designate significant differences: *P < 0.05 & **P < 0.01 vs. Vehicle group.

To further verify the cytoprotective mechanism of HZJW against gastric ulcer, NSAIDs-induced mucosal damage model was employed. It was observed that aspirin produced extensive necrosis of the gastric mucosa, whereas animals treated with HZJW (260, 520 and 1040 mg/kg) exhibited potent protection against aspirin-induced gastric ulceration in a dose-dependent manner. Orally applied HZJW significantly reduced the ulcer lesion index (35.40 ± 5.93; 31.30 ± 8.08, *P* < 0.05; 26.80 ± 8.27, *P* < 0.01) respectively, as compared to control group (41.60 ± 10.80). As well, ranitidine conferred prominent protection against ulcerogenesis (20.40 ± 6.93, *P* < 0.01). The extent of inhibitions for the respective doses employed was 14.90, 24.76, 35.58 and 50.96%, respectively. These results were summarized in Figure 2.

#### *In vitro* anti-*H*. *pylori* assay

The agar dilution method has been standardized as the reference method for minimum inhibitory concentration (MIC) determinations by the Clinical And Laboratory Standards Institute (CLSI). In the *in vitro* assay, MICs for HZJW and amoxicillin (positive control) were 125 and 0.12 μg/mL respectively. The *in vitro* anti-*H*. *pylori* potency of amoxicillin was far more pronounced than that of HZJW.

#### *In vivo* anti-*H*. *pylori* assay

**Figure 3:**
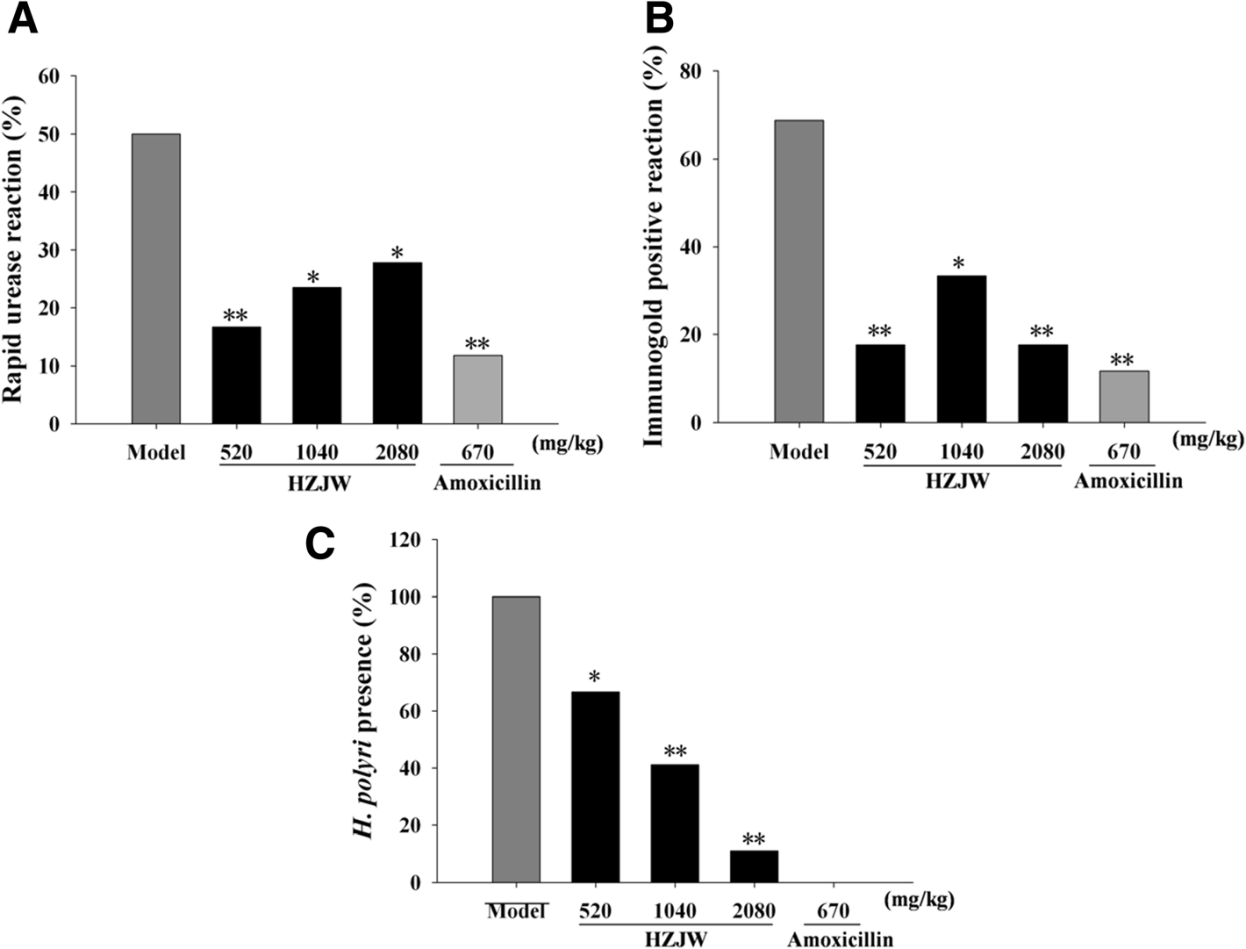
**Effect of HZJW and amoxicillin on the eradication of *Helicobacter pylori *via rapid urease test (A), immunogold test (B) and histopathological analysis (C)**. Vehicle group (Open column), amoxicillin group (670 mg/kg/d, grey bar), HZJW groups (520, 1040 and 2080 mg/kg/d, dark bars). *P < 0.05 & **P < 0.01 vs. Vehicle group.

Taking into consideration these data as well as the limitations of *in vitro* efficacy experimentation, we also evaluated the *in vivo* eradication potency of HZJW in previously described rapid screening mouse model [20]. In the rapid urease assay, all animals in control group were negative for urease reaction, whereas in *H*. *pylori*-inoculated group 50% were positive. Treatment with HZJW (520, 1040 and 2080 mg/kg) increased the negativity of the urease test in a dose-related manner, reaching 83.33% (*P* < 0.01), 76.5% and 72.2% (*P* < 0.05) respectively, in parallel to model animals. The test negativity rate was more prominent with HZJW treatment of lower dose. For the group given standard treatment--amoxicillin, negativity was 88.24% (*P* < 0.01), as shown in Figure 3A.

With respect to *H*. *pylori* urease Immunogold Test (Figure 3B), the infection rate for animals treated with HZJW of 520, 1040 and 2080 mg/kg was 17.65% (*P* < 0.01), 33.33% (*P* < 0.05) and 17.65% (*P* < 0.01) respectively, as compared with 68.75% of the model counterpart. The group treated with amoxicillin showed better results, in general, than those groups receiving HZJW with incidence rate of 11.76% (*P* < 0.01).

In relation to the presence of *H*. *pylori* in gastric mucosa, HZJW treatment depleted the number of viable *H*. *pylori* in gastric tissues of inoculated animals. According to the data obtained (Figure 3C), it was verified that those groups treated with 520, 1040 and 2080 mg/kg of HZJW presented a dose-dependent decrease in positive cases with carbolic acid & basic fuchsin staining, by 66.67% (*P* < 0.05), 41.18% (*P* < 0.001) and 11.11% (*P* < 0.01) respectively. Treatment with standard drugs caused complete elimination of *H*. *pylori*.

### Acute toxicity evaluation

The acute dose study provides a guideline for selecting doses for the subacute and chronic low-dose study, which may be more clinically relevant [21]. In the acute toxicity study, the oral LD_50_ of HZJW was over 18.0 g/kg by a single oral administration following fixed dose procedure. Animals did not manifest significant abnormal signs and alternations, behavioral changes, water or food consumption, body weight changes, or macroscopic findings at any time of observation. Autopsy results exhibited no significant change or lesion in the viscera of any animal.

### Survival and clinical signs

In the chronic oral toxicity assessment,animals treated with doses of 5000,2500 and 1000 mg/kg (equivalent to 50-, 25- and 10-fold human clinical doses, respectively) was observed after 26 weeks of daily administration of HZJW, as well as after the 4-week recovery period. No drug-related deaths were observed in any of the HZJW-treated groups. Six rats of both sexes experienced slight fur loss and the region of hair loss was limited on grasping area including shoulder and back neck and just partial loss. The symptom disappeared after certain period. No other test-article-related clinical signs were observed in rats dosed up to 5000 mg/kg with HZJW. No abnormal findings were observed during the ophthalmological examination in any of the groups (data not shown).

### Body weights, organ weights and feed consumption

**Figure 4:**
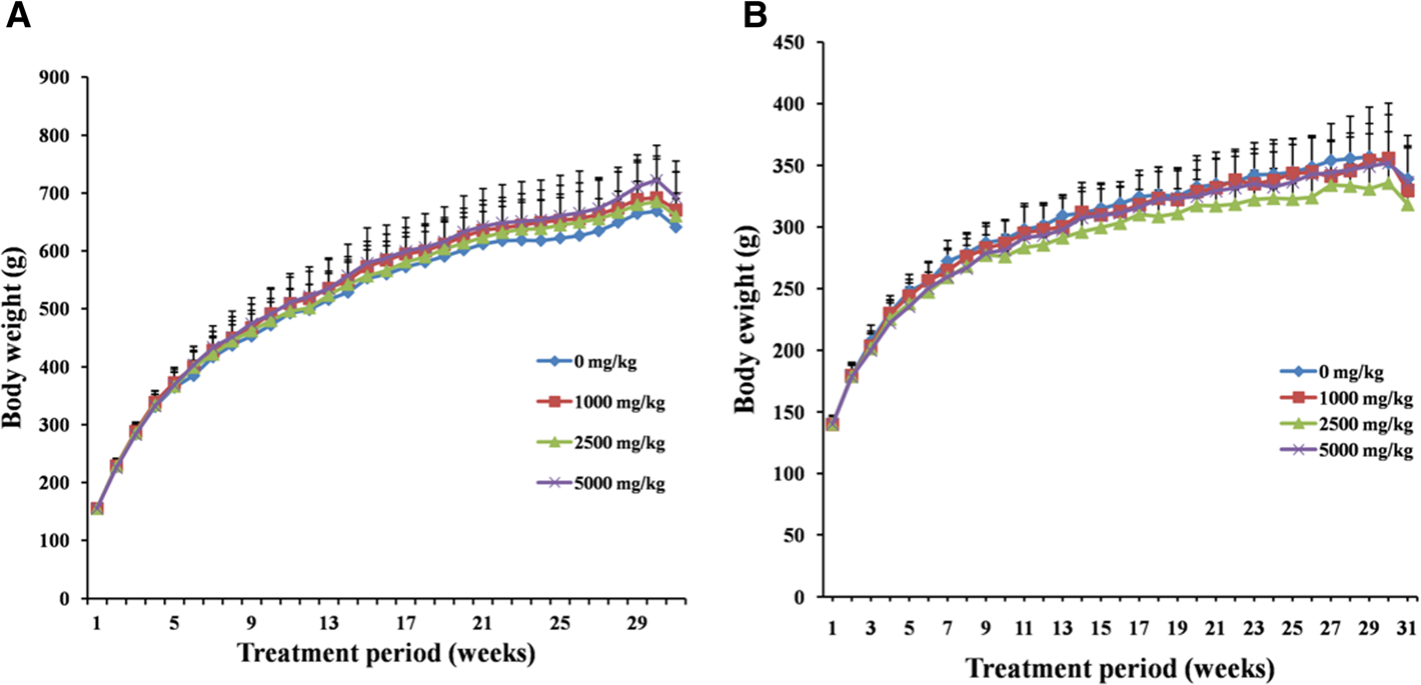
Mean body weight of male (A) and female (B) rats dosed with HZJW.

**Figure 5:**
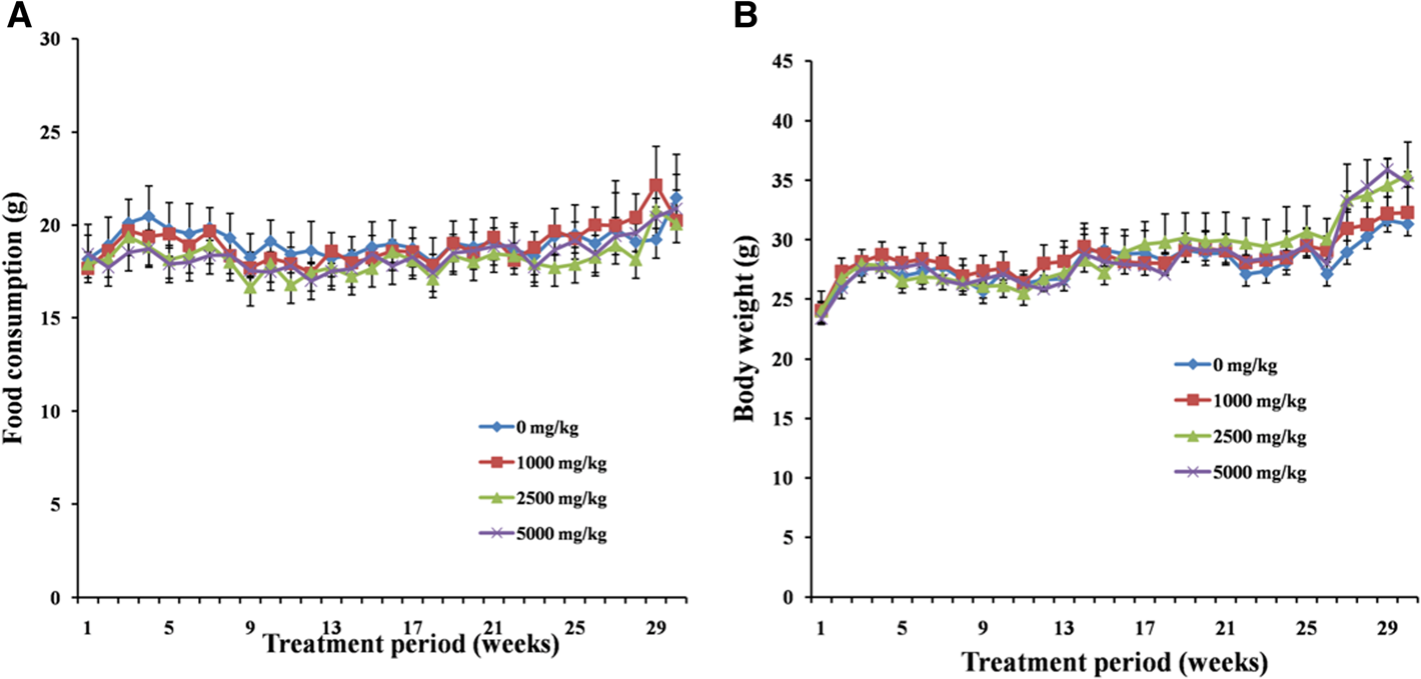
Mean food consumption by male (A) and female (B) rats treated with HZJW.

In all groups, body weight gradually increased for 6 months, and changes in body weight in HZJW treated groups relative to the control group were not significant during the experimental period (Figure 4). As expected, the rats gained weight with time. From the 1st week to the 30th week, body weight of animals in three treatment group showed no significant differences compared with that of control. No statistically significant differences in food (Figure 5) consumption were detected between the control and treatment group regardless of sex or recovery group. Absolute and relative organ weights of 26-week treated rats are shown in Table 2. Since these changes in organ weight by the end of week 26 and recovery were not accompanied by any relevant histopathological change. The organ weight changes were not considered to be of toxicological significance by HZJW treatment.

**Table 2:** Absolute & relative organ weights in rats orally treated with HZJW

**Parameter**	**Sex**	**Dose (mg/kg)**											
		**0 mg/kg**			**1000 mg/kg**			**2500 mg/kg**			**5000 mg/kg**		
		**13 wk**	**26 wk**	**Recovery**	**13 wk**	**26 wk**	**Recovery**	**13 wk**	**26 wk**	**Recovery**	**13wk**	**26 wk**	**Recovery**
Body weight	Male	486.2 ± 16.4	604.4 ± 70.0	641.3 ± 95.7	516.3 ± 101.3	634.6 ± 60.1	670.7 ± 65.7	482.3 ± 44.4	628.1 ± 67.7	660.4 ± 39.7	481.8 ± 39.5	637.8 ± 82.5	691.7 ± 63.8
	Female	291.3 ± 16.8	331.8 ± 27.2	338.9 ± 35.6	280.9 ± 17.0	333.4 ± 32.2	329.7 ± 34.7	266.0 ± 9.3	308.3 ± 25.7	318.4 ± 15.2	269.0 ± 36.4	322.2 ± 31.9	336.6 ± 29.5
Brain	Male	2.096 ± 0.064	2.240 ± 0.124	2.163 ± 0.103	2.214 ± 0.115	2.221 ± 0.095	2.191 ± 0.090	2.080 ± 0.117	2.281 ± 0.079	2.224 ± 0.092	2.092 ± 0.183	2.238 ± 0.089	2.261 ± 0.049
	Female	1.996 ± 0.066	2.021 ± 0.064	2.088 ± 0.096	1.947 ± 0.040	2.011 ± 0.064	2.028 ± 0.043	2.006 ± 0.046	2.008 ± 0.105	2.003 ± 0.063	1.985 ± 0.029	2.040 ± 0.060	2.029 ± 0.039
% to body weight	Male	0.431 ± .016	0.376 ± 0.054	0.344 ± 0.055	0.439 ± 0.067	0.352 ± 0.033	0.329 ± 0.034	0.434 ± 0.039	0.366 ± 0.036	0.338 ± 0.027	0.436 ± 0.043	0.356 ± 0.046	0.329 ± 0.030
	Female	0.687 ± 0.041	0.612 ± 0.041	0.621 ± 0.066	0.695 ± 0.045	0.608 ± 0.060	0.620 ± 0.065	0.754 ± 0.023*	0.655 ± 0.057	0.630 ± 0.019	0.747 ± 0.082	0.638 ± 0.065	0.606 ± 0.051
Thymus	Male	0.297 ± 0.097	0.270 ± 0.069	0.262 ± 0.063	0.351 ± 0.170	0.219 ± 0.106	0.250 ± 0.092	0.313 ± 0.043	0.223 ± 0.084	0.200 ± 0.038	0.270 ± 0.048	0.262 ± 0.056	0.224 ± 0.059
	Female	0.367 ± 0.057	0.245 ± 0.073	0.187 ± 0.057	0.325 ± 0.041	0.232 ± 0.038	0.225 ± 0.045	0.283 ± 0.053	0.226 ± 0.061	0.214 ± 0.071	0.304 ± 0.091	0.231 ± 0.063	0.218 ± 0.023
% to body weight	Male	0.061 ± 0.018	0.044 ± 0.009	0.041 ± 0.008	0.065 ± 0.019	0.034 ± 0.013*	0.037 ± 0.013	0.065 ± 0.011	0.035 ± 0.012	0.030 ± 0.005*	0.056 ± 0.008	0.041 ± 0.008	0.032 ± 0.006
	Female	0.126 ± 0.017	0.074 ± 0.023	0.055 ± 0.014	0.116 ± 0.015	0.070 ± 0.010	0.069 ± 0.015	0.106 ± 0.018	0.073 ± 0.017	0.067 ± 0.021	0.112 ± 0.026	0.071 ± 0.014	0.065 ± 0.009
Heart	Male	1.381 ± 0.111	1.625 ± 0.204	1.653 ± 0.259	1.494 ± 0.193	1.710 ± 0.229	1.613 ± 0.225	1.408 ± 0.156	1.702 ± 0.169	1.749 ± 0.138	1.467 ± 0.093	1.699 ± 0.182	1.912 ± 0.182
	Female	0.832 ± 0.042	1.100 ± 0.101	1.046 ± 0.138	0.917 ± 0.094	1.089 ± 0.109	1.013 ± 0.091	0.905 ± 0.066	1.024 ± 0.091	0.986 ± 0.069	0.969 ± 0.102	1.098 ± 0.136	1.014 ± 0.068
% to body weight	Male	0.284 ± 0.014	0.270 ± 0.029	0.258 ± 0.004	0.294 ± 0.037	0.271 ± 0.039	0.240 ± 0.015*	0.292 ± 0.021	0.272 ± 0.021	0.265 ± 0.020	0.306 ± 0.028	0.268 ± 0.016	0.277 ± 0.016
	Female	0.286 ± 0.012	0.333 ± 0.034	0.308 ± 0.010	0.326 ± 0.023*	0.328 ± 0.027	0.308 ± 0.016	0.341 ± 0.036*	0.334 ± 0.044	0.310 ± 0.016	0.362 ± 0.025**	0.341 ± 0.032	0.302 ± 0.018
Lung	Male	1.782 ± 0.201	1.831 ± 0.271	1.845 ± 0.237	1.862 ± 0.779	1.876 ± 0.246	1.910 ± 0.094	1.528 ± 0.103*	1.930 ± 0.088	1.916 ± 0.052	1.516 ± 0.066*	1.898 ± 0.127	2.099 ± 0.322
	Female	1.210 ± 0.061	1.434 ± 0.110	1.409 ± 0.098	1.506 ± 0.538	1.449 ± 0.168	1.28 ± 0.062*	1.210 ± 0.072	1.438 ± 0.166	1.419 ± 0.048	1.204 ± 0.192	1.443 ± 0.138	1.503 ± 0.152
% to body weight	Male	0.366 ± 0.038	0.304 ± 0.041	0.289 ± 0.025	0.354 ± 0.087	0.296 ± 0.034	0.287 ± 0.033	0.318 ± 0.021*	0.310 ± 0.029	0.291 ± 0.016	0.316 ± 0.026	0.300 ± 0.028	0.303 ± 0.035
	Female	0.416 ± 0.024	0.434 ± 0.036	0.418 ± 0.043	0.537 ± 0.189	0.438 ± 0.064	0.394 ± 0.041	0.456 ± 0.037	0.466 ± 0.036	0.446 ± 0.025	0.447 ± 0.021	0.450 ± 0.048	0.448 ± 0.041
Liver	Male	11.492 ± 1.894	12.852 ± 1.753	14.019 ± 2.841	12.564 ± 3.570	13.450 ± 1.903	14.001 ± 1.859	11.882 ± 1.267	14.860 ± 2.256*	15.212 ± 1.286	11.380 ± 0.892	16.043 ± 3.106*	16.306 ± 1.798
	Female	6.885 ± 0.495	8.213 ± 1.184	7.582 ± 0.862	7.057 ± 0.719	8.522 ± 0.984	7.761 ± 1.048	6.920 ± 0.305	8.136 ± 0.742	7.659 ± 0.794	7.356 ± 1.218	8.733 ± 0.742	7.594 ± 0.724
% to body weight	Male	2.356 ± 0.312	2.125 ± 0.125	2.180 ± 0.190	2.407 ± 0.256	2.117 ± 0.187	2.084 ± 0.143	2.461 ± 0.062	2.363 ± 0.209**	2.301 ± 0.061	2.366 ± 0.123	2.498 ± 0.193**	2.357 ± 0.164
	Female	2.369 ± 0.223	2.481 ± 0.355	2.239 ± 0.151	2.510 ± 0.189	2.561 ± 0.237	2.351 ± 0.132	2.605 ± 0.169	2.644 ± 0.205	2.402 ± 0.171	2.728 ± 0.105*	2.728 ± 0.300	2.258 ± 0.159
Spleen	Male	0.614 ± 0.042	0.799 ± 0.116	0.833 ± 0.115	0.639 ± 0.105	0.849 ± 0.110	0.795 ± 0.096	0.708 ± 0.166	0.810 ± 0.115	0.868 ± 0.133	0.673 ± 0.073	0.916 ± 0.101*	0.859 ± 0.104
	Female	0.431 ± 0.023	0.618 ± 0.149	0.582 ± 0.108	0.460 ± 0.054	0.544 ± 0.074	0.553 ± 0.060	0.478 ± 0.035	0.574 ± 0.115	0.545 ± 0.035	0.446 ± 0.081	0.518 ± 0.080	0.543 ± 0.057
% to body weight	Male	0.126 ± 0.006	0.133 ± 0.022	0.130 ± 0.009	0.126 ± 0.021	0.134 ± 0.009	0.118 ± 0.007	0.147 ± 0.030	0.129 ± 0.009	0.131 ± 0.018	0.140 ± 0.015	0.145 ± 0.015	0.124 ± 0.009
	Female	0.148 ± 0.009	0.187 ± 0.046	0.172 ± 0.026	0.164 ± 0.020	0.164 ± 0.021	0.168 ± 0.013	0.180 ± 0.016**	0.186 ± 0.033	0.171 ± 0.006	0.167 ± 0.028	0.162 ± 0.026	0.162 ± 0.018
Kidney	Male	2.832 ± 0.228	3.343 ± 0.322	3.406 ± 0.309	2.943 ± 0.433	3.421 ± 0.481	3.449 ± 0.451	2.766 ± 0.141	3.519 ± 0.316	3.856 ± 0.456	3.013 ± 0.428	3.622 ± 0.410	4.178 ± 0.503*
	Female	1.655 ± 0.139	1.896 ± 0.164	1.868 ± 0.199	1.568 ± 0.152	1.932 ± 0.175	1.914 ± 0.255	1.605 ± 0.045	1.978 ± 0.175	1.892 ± 0.111	1.626 ± 0.192	2.025 ± 0.164	1.857 ± 0.167
% to body weight	Male	0.582 ± 0.031	0.557 ± 0.056	0.537 ± 0.060	0.577 ± 0.070	0.540 ± 0.072	0.513 ± 0.029	0.577 ± 0.058	0.563 ± 0.048	0.584 ± 0.062	0.624 ± 0.059	0.572 ± 0.059	0.603 ± 0.026
	Female	0.569 ± 0.052	0.574 ± 0.058	0.552 ± 0.031	0.558 ± 0.048	0.582 ± 0.054	0.581 ± 0.049	0.604 ± 0.027	0.643 ± 0.049**	0.594 ± 0.024*	0.606 ± 0.029*	0.632 ± 0.056	0.552 ± 0.034
Adrenal grand	Male	0.068 ± 0.012	0.050 ± 0.006	0.062 ± 0.009	0.059 ± 0.013	0.056 ± 0.007	0.051 ± 0.005*	0.062 ± 0.012	0.055 ± 0.009	0.057 ± 0.005	0.053 ± 0.009*	0.056 ± 0.008	0.065 ± 0.010
	Female	0.066 ± 0.007	0.063 ± 0.009	0.067 ± 0.009	0.061 ± 0.009	0.063 ± 0.008	0.055 ± 0.006*	0.066 ± 0.006	0.065 ± 0.012	0.064 ± 0.011	0.061 ± 0.008	0.069 ± 0.009	0.057 ± 0.010
% to body weight	Male	0.014 ± 0.002	0.008 ± 0.002	0.010 ± 0.001	0.012 ± 0.004	0.009 ± 0.001	0.008 ± 0.001*	0.013 ± 0.003	0.009 ± 0.002	0.009 ± 0.001	0.011 ± 0.002	0.009 ± 0.002	0.009 ± 0.001
	Female	0.023 ± 0.002	0.019 ± 0.003	0.020 ± 0.002	0.022 ± 0.003	0.019 ± 0.003	0.017 ± 0.001*	0.025 ± 0.003	0.021 ± 0.004	0.020 ± 0.004	0.023 ± 0.004	0.022 ± 0.003	0.017 ± 0.003
Testis(Rt.)/ Ovary (Lt.)	Male	3.391 ± 0.243	3.669 ± 0.398	3.767 ± 0.306	3.607 ± 0.142	3.606 ± 0.788	3.666 ± 0.315	3.324 ± 0.689	3.831 ± 0.472	3.822 ± 0.331	3.594 ± 0.362	3.632 ± 0.851	4.047 ± 0.343
	Female	3.594 ± 0.362	0.146 ± 0.030	0.164 ± 0.049	0.145 ± 0.017	0.157 ± 0.038	0.157 ± 0.047	0.149 ± 0.017	0.156 ± 0.022	0.152 ± 0.016	0.148 ± 0.018	0.166 ± 0.036	0.164 ± 0.017
% to body weight	Male	0.698 ± 0.052	0.610 ± 0.054	0.593 ± 0.050	0.716 ± 0.109	0.567 ± 0.109	0.548 ± 0.033	0.684 ± 0.095	0.613 ± 0.079	0.579 ± 0.032	0.751 ± 0.114	0.571 ± 0.128	0.587 ± 0.046
	Female	0.046 ± 0.007	0.044 ± 0.010	0.048 ± 0.014	0.051 ± 0.005*	0.048 ± 0.013	0.049 ± 0.015	0.056 ± 0.007*	0.051 ± 0.006	0.048 ± 0.006	0.055 ± 0.002	0.052 ± 0.010	0.049 ± 0.006
Epididymis (Lt.)/Uterus	Male	1.264 ± 0.099	1.542 ± 0.205	1.555 ± 0.142	1.440 ± 0.207	1.547 ± 0.329	1.562 ± 0.403	1.322 ± 0.170	1.550 ± 0.306	1.575 ± 0.170	1.431 ± 0.080*	1.511 ± 0.271	1.669 ± 0.262
	Female	0.649 ± 0.173	0.803 ± 0.289	0.883 ± 0.114	0.879 ± 0.283	0.991 ± 0.340	0.970 ± 0.274	0.715 ± 0.195	0.907 ± 0.213	0.691 ± 0.152	0.752 ± 0.338	0.770 ± 0.264	0.893 ± 0.365
% to body weight	Male	0.260 ± 0.021	0.260 ± 0.054	0.245 ± 0.026	0.285 ± 0.050	0.244 ± 0.047	0.231 ± 0.043	0.273 ± 0.015	0.248 ± 0.050	0.239 ± 0.032	0.299 ± 0.035	0.240 ± 0.053	0.241 ± 0.029
	Female	0.225 ± 0.070	0.245 ± 0.092	0.264 ± 0.048	0.315 ± 0.108	0.299 ± 0.102	0.296 ± 0.084	0.269 ± 0.074	0.298 ± 0.082	0.217 ± 0.044	0.272 ± 0.084	0.240 ± 0.082	0.271 ± 0.125

Note: Data represents body weights right before necropsy, after fasting; Values are mean ± S.D. (*n* = 5 (4 weeks or recovery), 10 (26 weeks), recovery (30 weeks)/sex/dose). * *P* < 0.05 & ** *P* < 0.01 compared with normal group.

### Hematological parameters

Hematopoietic indices have been reported to be very sensitive to toxic compounds and serve as important index of physiologic and pathologic status for both animals and humans [22]. The effect of HZJW on the hematological parameters of the experimental and control rats is presented in Table 3. The results indicated that all hematological parameters measured remained within the physiological range throughout the 30-week experimental period. There were no significant changes in mean corpuscular volume, red cell distribution width, platelets, platelet distribution width, prothrombin time, eosinophils, basophils, monocytes, and reticulocytes values between the control and treated animals (main groups). Despite a significant change was observed in some parameters (hemoglobin, mean corpuscular hemoglobin, mean corpuscular hemoglobin concentration, mean platelet volume, white blood cell, lymphocytes, neutrophils), the above parameters remained within the range of normal physiological variation and there was no dose response relationship.

**Table 3:** Hematological values in rats orally treated with HZJW

**Parameter unit sex**			**Hematological values**											
			**0 mg/kg**			**1000 mg/kg**			**2500 mg/kg**			**5000 mg/kg**		
			**13 wk**	**26 wk**	**Recovery**	**13 wk**	**26 wk**	**Recovery**	**13 wk**	**26 wk**	**Recovery**	**13wk**	**26 wk**	**Recovery**
RBC	10^12^/L	Male	9.18 ± 0.31	8.86 ± 0.47	8.65 ± 0.32	9.04 ± 0.33	8.72 ± 0.40	8.83 ± 0.45	9.27 ± 0.29	8.21 ± 0.71	8.48 ± 0.35	8.99 ± 0.80	8.94 ± 0.50	9.07 ± 0.71
		Female	8.11 ± 0.28	7.90 ± 0.49	7.59 ± 0.55	8.30 ± 0.28	7.85 ± 0.69	8.01 ± 0.72	8.37 ± 0.79	7.84 ± 0.49	7.65 ± 0.41	8.58 ± 0.66	6.96 ± 1.47	7.90 ± 0.55
HGB	g/L	Male	174 ± 6	168 ± 6	170 ± 7	164 ± 7	166 ± 3	166 ± 3	171 ± 5	157 ± 11**	163 ± 5	168 ± 6	162 ± 6*	174 ± 8
		Female	158 ± 2	162 ± 7	159 ± 10	163 ± 4	156 ± 12	167 ± 11	166 ± 10	155 ± 8	160 ± 10	166 ± 12	137 ± 29*	162 ± 4
HCT	%	Male	45.3 ± 1.3	43.7 ± 1.8	44.1 ± 1.8	43.9 ± 1.2	44.1 ± 1.1	43.3 ± 0.9	44.9 ± 1.3	42.2 ± 3.2	43.0 ± 1.4	44.4 ± 2.5	45.0 ± 2.4	46.5 ± 2.4
		Female	40.8 ± 1.2	42.2 ± 2.3	40.7 ± 2.8	41.9 ± 1.2	41.6 ± 2.9	44.3 ± 3.1	42.2 ± 3.3	41.7 ± 2.4	41.0 ± 3.0	42.7 ± 2.6	37.8 ± 7.8	41.0 ± 1.7
MCV	fL	Male	49.3 ± 0.7	49.3 ± 1.2	51.0 ± 1.9	48.6 ± 1.7	50.6 ± 1.5	49.1 ± 1.6	48.4 ± 0.8	51.5 ± 1.8	50.7 ± 2.1	49.6 ± 3.3	50.3 ± 1.7	51.4 ± 1.5
		Female	50.3 ± 1.4	53.5 ± 1.3	53.7 ± 2.4	50.5 ± 1.7	53.0 ± 1.7	55.4 ± 1.5	50.4 ± 0.9	53.2 ± 1.7	53.6 ± 1.3	49.8 ± 1.1	54.4 ± 1.6	52.0 ± 1.7
MCH	pg	Male	19.0 ± 0.2	19.0 ± 0.6	19.7 ± 0.8	18.1 ± 0.5	19.1 ± 0.7	18.8 ± 0.9	18.4 ± 0.3	19.1 ± 0.9	19.3 ± 0.7	18.8 ± 1.4	18.1 ± 0.7*	19.2 ± 0.7
		Female	19.5 ± 0.7	20.6 ± 0.5	21.0 ± 0.8	19.7 ± 0.6	19.9 ± 0.7**	20.9 ± 0.8	19.9 ± 0.9	19.8 ± 0.6*	20.9 ± 0.5	19.4 ± 1.0	19.7 ± 0.9**	20.6 ± 0.9
MCHC	g/L	Male	385 ± 6	385 ± 7	386 ± 9	373 ± 9	376 ± 7*	383 ± 6	381 ± 4	371 ± 13*	380 ± 5	378 ± 10	361 ± 8*	374 ± 4*
		Female	387 ± 8	385 ± 6	391 ± 8	389 ± 3	376 ± 7*	378 ± 8	395 ± 12	373 ± 6*	389 ± 8	389 ± 17	362 ± 16*	395 ± 7
RDW	%	Male	13.6 ± 0.5	13.3 ± 0.5	13.5 ± 0.4	13.7 ± 0.2	13.3 ± 0.3	13.4 ± 0.3	13.5 ± 0.3	13.3 ± 0.5	13.4 ± 0.5	13.7 ± 0.3	13.4 ± 0.4	13.4 ± 0.7
		Female	13.1 ± 0.3	12.8 ± 0.3	12.6 ± 0.3	13.0 ± 0.3	12.7 ± 0.3	12.5 ± 0.2	12.9 ± 0.3	12.6 ± 0.3	12.9 ± 0.2	12.8 ± 0.3	13.0 ± 0.4	12.6 ± 0.5
PLT	10^9^/L	Male	823 ± 87	906 ± 48	881 ± 107	797 ± 106	831 ± 71	906 ± 76	816 ± 39	819 ± 74	872 ± 42	795 ± 30	854 ± 69	940 ± 99
		Female	763 ± 55	756 ± 98	828 ± 60	859 ± 122	816 ± 62	936 ± 72	810 ± 45	792 ± 94	839 ± 91	848 ± 86	696 ± 271	865 ± 76
MPV	fL	Male	3.3 ± 0.4	3.7 ± 0.5	4.2 ± 0.7	3.3 ± 0.4	3.7 ± 0.4	4.2 ± 0.4	3.4 ± 0.4	3.9 ± 0.5	4.2 ± 0.4	3.5 ± 0.4	4.7 ± 0.5**	4.5 ± 0.6
		Female	3.4 ± 0.4	3.7 ± 0.5	4.1 ± 0.9	3.4 ± 0.4	4.0 ± 0.5	4.5 ± 0.2	3.5 ± 0.2	4.0 ± 0.6	4.4 ± 0.5	3.5 ± 0.4	4.6 ± 1.6	4.0 ± 0.2
PDW	%	Male	14.8 ± 0.5	14.4 ± 0.3	14.3 ± 0.6	15.3 ± 0.4	14.3 ± 0.6	13.9 ± 0.2	14.9 ± 0.5	14.3 ± 0.4	14.4 ± 0.5	15.2 ± 1.0	14.3 ± 0.4	14.6 ± 0.6
		Female	15.0 ± 0.5	14.7 ± 0.3	14.4 ± 0.6	14.9 ± 0.4	14.7 ± 0.4	15.1 ± 0.8	15.3 ± 0.8	14.6 ± 0.6	14.5 ± 0.7	15.3 ± 0.6	15.2 ± 1.9	14.1 ± 0.2
PT	s	Male	14.4 ± 1.8	19.0 ± 5.8	15.1 ± 0.9	18.1 ± 3.9	17.1 ± 4.4	15.1 ± 0.4	13.5 ± 0.4	15.2 ± 3.2	14.7 ± 0.4	15.2 ± 2.6	15.7 ± 3.4	14.5 ± 0.6
		Female	12.7 ± 0.6	13.4 ± 0.6	14.5 ± 0.5	12.4 ± 0.5	13.3 ± 0.5	14.4 ± 0.6	12.5 ± 0.3	13.7 ± 0.7	13.9 ± 0.5	12.5 ± 0.8	13.2 ± 1.0	14.1 ± 1.2
WBC	10^9^/L	Male	6.4 ± 0.4	4.6 ± 1.1	4.7 ± 1.6	8.5 ± 3.4	6.6 ± 1.2**	5.2 ± 2.1	10.5 ± 2.7**	6.1 ± 2.3	5.2 ± 0.9	11.9 ± 2.2*	6.5 ± 2.2*	5.5 ± 1.2
		Female	4.3 ± 1.2	3.1 ± 0.9	2.3 ± 0.6	6.0 ± 1.4	2.8 ± 0.8	2.4 ± 0.5	6.6 ± 2.5	3.1 ± 0.8	2.3 ± 0.6	6.9 ± 1.3	2.7 ± 1.2	2.7 ± 0.6
LYM	10^9^/L	Male	5.0 ± 0.4	3.9 ± 0.9	4.1 ± 1.3	6.7 ± 3.1	5.3 ± 0.9**	4.4 ± 1.8	7.6 ± 1.8*	4.8 ± 1.8	4.3 ± 0.6	9.3 ± 2.1**	4.9 ± 1.6	4.4 ± 0.9
		Female	3.5 ± 1.1	2.5 ± 0.7	1.9 ± 0.5	4.8 ± 0.8	2.2 ± 0.6	2.0 ± 0.4	5.3 ± 2.0	2.5 ± 0.6	1.9 ± 0.5	5.8 ± 1.4*	2.2 ± 1.0	2.2 ± 0.4
MON	10^9^/L	Male	0.1 ± 0.0	0.0 ± 0.1	0.0 ± 0.0	0.2 ± 0.1	0.1 ± 0.0	0.0 ± 0.0	0.2 ± 0.2	0.1 ± 0.1	0.0 ± 0.0	0.3 ± 0.1	0.1 ± 0.1	0.1 ± 0.0
		Female	0.1 ± 0.1	0.1 ± 0.2	0.0 ± 0.0	0.1 ± 0.1	0.0 ± 0.0	0.0 ± 0.0	0.2 ± 0.1	0.0 ± 0.0	0.0 ± 0.0	0.2 ± 0.1	0.0 ± 0.0	0.0 ± 0.0
NE	10^9^/L	Male	1.2 ± 0.3	0.6 ± 0.2	0.6 ± 0.3	1.6 ± 0.5	1.2 ± 0.3**	0.8 ± 0.3	2.6 ± 0.8**	1.2 ± 0.5**	0.9 ± 0.2	2.2 ± 0.4**	1.5 ± 0.7**	1.1 ± 0.3
		Female	0.7 ± 0.3	0.5 ± 0.2	0.3 ± 0.1	1.0 ± 0.7	0.6 ± 0.2	0.4 ± 0.1	1.1 ± 0.7	0.6 ± 0.1	0.4 ± 0.1	0.9 ± 0.3	0.5 ± 0.2	0.5 ± 0.1
EOS	10^9^/L	Male	0.0 ± 0.1	0.0 ± 0.0	0.0 ± 0.0	0.0 ± 0.0	0.0 ± 0.0	0.0 ± 0.0	0.0 ± 0.0	0.0 ± 0.0	0.0 ± 0.0	0.1 ± 0.1	0.0 ± 0.0	0.0 ± 0.0
		Female	0.0 ± 0.1	0.0 ± 0.0	0.0 ± 0.0	0.1 ± 0.0	0.0 ± 0.0	0.0 ± 0.0	0.0 ± 0.1	0.0 ± 0.0	0.0 ± 0.0	0.0 ± 0.1	0.0 ± 0.0	0.0 ± 0.0
BAS	10^9^/L	Male	0.0 ± 0.0	0.0 ± 0.0	0.0 ± 0.0	0.0 ± 0.0	0.0 ± 0.0	0.0 ± 0.0	0.0 ± 0.0	0.0 ± 0.0	0.0 ± 0.0	0.0 ± 0.0	0.0 ± 0.0	0.0 ± 0.0
		Female	0.0 ± 0.0	0.0 ± 0.0	0.0 ± 0.0	0.0 ± 0.0	0.0 ± 0.0	0.0 ± 0.0	0.0 ± 0.0	0.0 ± 0.0	0.0 ± 0.0	0.0 ± 0.0	0.0 ± 0.0	0.0 ± 0.0
LYM	%	Male	77.7 ± 4.5	84.9 ± 0.9	86.7 ± 1.0	77.5 ± 5.2	80.7 ± 2.7**	83.4 ± 0.6**	72.8 ± 4.0	77.3 ± 2.4*	80.8 ± 1.8**	78.0 ± 4.7	75.9 ± 3.6**	79.0 ± 3.1**
		Female	80.2 ± 6.1	80.0 ± 2.1	85.7 ± 0.7	80.3 ± 7.7	77.5 ± 3.7	83.9 ± 1.4*	79.8 ± 7.8	77.2 ± 2.3*	81.7 ± 2.6*	81.7 ± 6.2	78.9 ± 4.9	80.0 ± 2.0**
MON	%	Male	1.8 ± 0.3	1.1 ± 0.5	0.4 ± 0.1	1.9 ± 1.1	1.1 ± 0.4	0.5 ± 0.3	2.3 ± 0.7	1.4 ± 0.7	0.5 ± 0.2	2.7 ± 0.6	1.3 ± 0.5	1.1 ± 0.4
		Female	1.5 ± 0.5	2.4 ± 4.3	0.3 ± 0.2	2.6 ± 0.5	0.9 ± 0.4	0.3 ± 0.3	2.7 ± 1.1	1.2 ± 0.6	0.6 ± 0.4	3.0 ± 0.6	0.9 ± 0.6	0.5 ± 0.3
NE	%	Male	19.5 ± 4.8	13.4 ± 0.7	12.3 ± 1.1	20.0 ± 4.8	17.6 ± 3.0**	15.8 ± 0.6	24.5 ± 4.0	20.6 ± 2.6*	18.1 ± 1.7	18.7 ± 4.3	22.0 ± 3.6**	19.3 ± 3.4
		Female	16.8 ± 6.3	16.9 ± 4.4	13.8 ± 0.7	15.5 ± 7.9	20.7 ± 3.5	15.2 ± 1.5	16.6 ± 8.7	20.6 ± 2.7*	16.7 ± 2.6	14.2 ± 6.0	19.3 ± 4.1	18.7 ± 1.8
EOS	%	Male	0.7 ± 0.3	0.4 ± 0.3	0.4 ± 0.4 0	0.4 ± 0.3	0.3 ± 0.2	0.2 ± 0.1	0.3 ± 0.2	0.4 ± 0.3	0.4 ± 0.3	0.5 ± 0.2	0.5 ± 0.4	0.4 ± 0.2
		Female	1.2 ± 0.2	0.5 ± 0.2	0.1 ± 0.1	1.0 ± 0.3	0.5 ± 0.2	0.5 ± 0.1	0.7 ± 0.5	0.7 ± 0.5	0.8 ± 0.6	0.8 ± 0.4	0.8 ± 0.5	0.5 ± 0.4
BAS	%	Male	0.3 ± 0.1	0.3 ± 0.2	0.2 ± 0.1	0.2 ± 0.2	0.3 ± 0.2	0.2 ± 0.1	0.1 ± 0.1	0.3 ± 0.2	0.1 ± 0.1	0.1 ± 0.1	0.3 ± 0.1	0.2 ± 0.0
		Female	0.2 ± 0.2	0.3 ± 0.3	0.1 ± 0.1	0.5 ± 0.2	0.4 ± 0.3	0.1 ± 0.1	0.2 ± 0.3	0.2 ± 0.2	0.2 ± 0.3	0.3 ± 0.1	0.1 ± 0.2	0.2 ± 0.3
RET	‰	Male	11 ± 4	21 ± 5	10 ± 3	14 ± 6	18 ± 3	12 ± 2	16 ± 3*	18 ± 7	11 ± 4	14 ± 4	21 ± 5	11 ± 4
		Female	18 ± 4	21 ± 4	10 ± 3	19 ± 5	22 ± 4	17 ± 2	18 ± 4	18 ± 6	11 ± 3	14 ± 4	28 ± 7	10 ± 4

Note: Red blood cell (RBC), hemoglobin (HGB), hematocrit (HCT), mean corpuscular volume (MCV), mean corpuscular HGB (MCH), mean corpuscular HGB concentration (MCHC), red cell distribution width (RDW), platelets (PLT), mean platelet volume (MPV), platelet distribution width (PDW), white blood cell (WBC) counts, reticulocytes (RET), neutrophils (NE), lymphocytes (LYM), monocytes (MON), eosinophils (EOS), and basophils (BAS) prothrombin time (PT). Values are mean ± S.D. (*n* = 5 (4 weeks), 10 (26 weeks) or recovery (30 weeks)/sex/dose). * *P* < 0.05 & ** *P* < 0.01 compared with normal group.

### Blood biochemistry

The data for the biochemical parameters in the treated and control rats are presented in Table 4. All the parameters remained within the physiological range throughout the 30-week experimental period. No statistically significant differences were observed amongst the blood chemistry, including alkaline phosphatase activity, total cholesterol, urea, creatine phosphokinase activity, sodium ions, potassium ions and chlorideions between the control and treated animals. Despite some parameters experienced significant variation (glucose, triglycerides, aspartate aminotransferase activity, alanine aminotransferase activity, total protein, total bilirubin, direct bilirubin, alkaline phosphatase activity, albumin and urea), there were no dose response relationships and all of above parameters remained within the physiological range.

**Table 4:** Serum biochemical values in rats orally treated with HZJW

**Parameter unit sex**			**Dose (mg/kg)**											
			**0 mg/kg**			**1000 mg/kg**			**2500 mg/kg**			**5000 mg/kg**		
			**13 wk**	**26 wk**	**Recovery**	**13 wk**	**26 wk**	**Recovery**	**13 wk**	**26 wk**	**Recovery**	**13wk**	**26 wk**	**Recovery**
ALT	U/L	Male	40 ± 3	39 ± 11	34 ± 8	31 ± 8	42 ± 11	34 ± 12	41 ± 4	34 ± 10	47 ± 14	41 ± 10	30 ± 6	37 ± 9
		Female	43 ± 8	44 ± 22	32 ± 8	29 ± 8*	59 ± 32	39 ± 26	33 ± 9	39 ± 23	41 ± 43	27 ± 4**	28 ± 6	28 ± 7
AST	U/L	Male	161 ± 6	109 ± 28	80 ± 15	114 ± 24**	113 ± 36	91 ± 40	124 ± 13**	83 ± 13	112 ± 21	121 ± 20**	87 ± 25	105 ± 21
		Female	161 ± 35	122 ± 81	78 ± 17	113 ± 18*	155 ± 72	100 ± 42	127 ± 27	104 ± 45	86 ± 71	98 ± 14**	81 ± 20	65 ± 12
LP	U/L	Male	90 ± 10	106 ± 38	77 ± 27	105 ± 29	70 ± 15	80 ± 43	106 ± 27	79 ± 21	84 ± 14	98 ± 22	68 ± 20	93 ± 15
		Female	61 ± 19	51 ± 15	59 ± 17	60 ± 11	46 ± 18	48 ± 17	55 ± 23	54 ± 11	41 ± 22	55 ± 23	44 ± 15	57 ± 19
GLU	mmol/L	Male	7.26 ± 0.73	6.07 ± 0.78	7.89 ± 1.27	6.99 ± 1.49	6.69 ± 0.75	7.85 ± 1.03	6.27 ± 0.68	7.84 ± 1.30	7.48 ± 0.84	7.38 ± 0.78	8.39 ± 0.78**	8.55 ± 1.22
		Female	7.60 ± 0.95	6.57 ± 0.80	7.10 ± 0.36	7.04 ± 0.83	6.81 ± 0.60	6.61 ± 0.56	6.35 ± 1.27	6.45 ± 0.63	7.02 ± 0.43	7.66 ± 1.33	7.48 ± 1.05	7.30 ± 0.35
CHOL	mmol/L	Male	1.20 ± 0.26	1.53 ± 0.28	1.63 ± 0.21	1.34 ± 0.33	1.44 ± 0.35	1.41 ± 0.10	1.58 ± 0.26	1.53 ± 0.36	1.73 ± 0.13	1.81 ± 0.36	1.46 ± 0.10	1.35 ± 0.21
		Female	1.77 ± 0.38	2.20 ± 0.38	1.97 ± 0.47	2.14 ± 0.31	2.33 ± 0.42	2.46 ± 1.10	2.23 ± 0.39	2.25 ± 0.56	2.20 ± 0.46	2.07 ± 0.38	2.08 ± 0.44	1.92 ± 0.53
TG	mmol/L	Male	0.50 ± 0.06	0.66 ± 0.20	0.74 ± 0.73	0.90 ± 0.45	0.84 ± 0.36	0.85 ± 0.27	0.84 ± 0.36	0.92 ± 0.39	0.65 ± 0.16	0.74 ± 0.03**	1.01 ± 0.39*	0.74 ± 0.41
		Female	0.62 ± 0.08	1.18 ± 0.62	0.70 ± 0.46	0.70 ± 0.13	0.92 ± 0.50	0.85 ± 0.28	0.58 ± 0.11	0.51 ± 0.12	0.61 ± 0.25	0.84 ± 0.32	0.75 ± 0.25	0.59 ± 0.20
TP	g/L	Male	5.29 ± 0.12	5.36 ± 0.29	5.54 ± 0.25	5.34 ± 0.25	5.50 ± 0.47	5.45 ± 0.18	5.70 ± 0.19**	5.37 ± 0.51	5.50 ± 0.19	5.84 ± 0.13**	5.45 ± 0.38	5.67 ± 0.22
		Female	5.93 ± 0.22	6.34 ± 0.27	6.32 ± 0.42	6.31 ± 0.48	6.72 ± 0.50	6.81 ± 0.33	6.39 ± 0.27*	6.37 ± 0.43	6.32 ± 0.26	6.48 ± 0.26**	6.49 ± 0.50	6.22 ± 0.27
ALB	g/L	Male	3.02 ± 0.08	2.83 ± 0.34	3.04 ± 0.15	2.99 ± 0.21	2.92 ± 0.40	3.12 ± 0.13	3.33 ± 0.15**	2.86 ± 0.45	3.18 ± 0.06	3.37 ± 0.11**	2.95 ± 0.41	3.09 ± 0.13
		Female	3.50 ± 0.22	3.63 ± 0.30	3.78 ± 0.35	3.69 ± 0.28	3.83 ± 0.31	4.00 ± 0.15	3.65 ± 0.19	3.64 ± 0.30	3.70 ± 0.26	3.87 ± 0.22*	3.61 ± 0.38	3.71 ± 0.17
T-BIL	μmol/L	Male	3.4 ± 0.4	2.9 ± 1.6	3.0 ± 2.5	3.8 ± 0.9	3.0 ± 0.7	2.4 ± 0.5	3.1 ± 0.9	3.1 ± 0.6	2.0 ± 0.4	3.5 ± 0.8	2.9 ± 1.2	2.6 ± 1.1
		Female	3.9 ± 0.5	4.1 ± 1.4	3.0 ± 0.8	3.0 ± 0.7	3.6 ± 0.8	3.7 ± 0.9	3.7 ± 1.0	2.7 ± 0.6*	2.7 ± 0.5	3.1 ± 0.7	3.9 ± 0.8	2.8 ± 0.5
D-BIL	μmol/L	Male	2.4 ± 0.4	1.4 ± 0.5	1.8 ± 0.6	2.2 ± 0.6	1.2 ± 0.5	1.7 ± 0.2	1.2 ± 0.4	1.0 ± 0.4	1.4 ± 0.1	1.3 ± 0.6	1.1 ± 0.9	1.6 ± 0.5
		Female	2.5 ± 0.1	2.0 ± 1.0	1.9 ± 0.4	1.5 ± 0.3	1.5 ± 0.6	2.5 ± 0.4	1.8 ± 0.7	0.9 ± 0.4**	1.9 ± 0.3	0.9 ± 0.2	1.4 ± 0.9	1.8 ± 0.3
Cre	μmol/L	Male	70.3 ± 4.6	63.6 ± 9.5	65.2 ± 10.3	74.7 ± 9.0	64.8 ± 11.9	56.1 ± 4.8	80.3 ± 10.0	62.9 ± 10.3	73.3 ± 11.8	80.5 ± 4.6	64.7 ± 4.1	76.2 ± 2.7
		Female	78.8 ± 5.9	68.2 ± 8.1	67.3 ± 7.2	79.4 ± 5.2	66.9 ± 5.1	65.5 ± 3.4	82.6 ± 6.4	67.1 ± 5.7	63.2 ± 1.1	78.8 ± 8.9	71.4 ± 7.7	66.7 ± 5.3
CPK	U/L	Male	3214 ± 597	335 ± 141	183 ± 47	767 ± 931	325 ± 324	150 ± 46	404 ± 61	218 ± 119	301 ± 38	509 ± 220	245 ± 111	362 ± 130
		Female	2032 ± 1363	244 ± 61	168 ± 89	801 ± 616	248 ± 173	231 ± 123	488 ± 143	277 ± 133	120 ± 54	264 ± 51	158 ± 44	111 ± 34
Ure	mmol/L	Male	4.85 ± 0.26	5.77 ± 0.82	5.17 ± 1.00	4.57 ± 0.44	5.53 ± 1.22	5.35 ± 0.66	4.23 ± 0.65	5.88 ± 0.73	5.93 ± 0.39	4.33 ± 0.43	5.66 ± 1.08	6.02 ± 1.44
		Female	5.44 ± 0.49	6.08 ± 0.73	6.32 ± 0.89	5.04 ± 0.51	6.04 ± 0.92	6.13 ± 1.01	5.42 ± 0.59	6.72 ± 1.11	6.45 ± 0.67	5.71 ± 0.87	6.74 ± 1.31**	6.24 ± 1.12
Na^+^	mmol/L	Male	141.3 ± 0.7	141.8 ± 1.4	142.3 ± 0.2	141.0 ± 0.4	142.4 ± 1.6	142.3 ± 1.2	140.7 ± 1.3	141.2 ± 0.7	141.9 ± 1.5	139.8 ± 0.7	141.1 ± 1.4	141.1 ± 0.8
		Female	141.6 ± 0.9	140.0 ± 1.9	141.8 ± 0.9	140.9 ± 1.0	140.9 ± 0.9	143.0 ± 0.6	140.3 ± 1.0	141.0 ± 1.7	142.6 ± 1.4	139.4 ± 1.0	139.8 ± 0.8	142.5 ± 0.7
K^+^	mmol/L	Male	4.81 ± 0.27	3.95 ± 0.27	3.65 ± 0.20	5.02 ± 0.43	4.08 ± 0.64	3.50 ± 0.28	5.16 ± 0.32	4.15 ± 0.70	3.90 ± 0.20	5.13 ± 0.12	3.92 ± 0.56	4.09 ± 0.27
		Female	4.43 ± 0.25	3.86 ± 0.33	3.43 ± 0.36	4.80 ± 0.34	3.57 ± 0.20	3.42 ± 0.08	4.60 ± 0.37	3.59 ± 0.27	3.46 ± 0.21	4.99 ± 0.54	4.57 ± 2.02	3.33 ± 0.31
Cl^-^	mmol/L	Male	111.1 ± 0.4	108.7 ± 2.9	107.0 ± 2.2	110.3 ± 0.9	106.7 ± 1.5	109.5 ± 1.6	108.2 ± 1.3	108.0 ± 2.9	107.2 ± 0.5	106.4 ± 1.6	107.6 ± 2.2	106.5 ± 1.2
		Female	110.3 ± 3.3	108.9 ± 2.3	109.8 ± 1.6	109.7 ± 2.1	110.7 ± 1.4	109.8 ± 0.8	109.8 ± 1.6	109.2 ± 2.8	109.6 ± 1.2	107.3 ± 1.2	106.7 ± 1.6	109.1 ± 1.1

Note: Aspartate aminotransferase activity (AST), alanine aminotransferase activity (ALT), alkaline phosphatase activity (ALP), albumin (ALB), total protein (TP), glucose (GLU), total cholesterol (CHOL), creatine phosphokinase activity (CPK), total bilirubin (T-BIL), direct bilirubin (D-BIL), creatinine (CRE), triglycerides (TG), urea (Ure), sodium ions (Na^+^), potassium ions (K^+^), chlorideions (Cl^-^). Values are mean ± S.D. (*n* = 5 (4 weeks), 10 (26 weeks) or recovery (30 weeks)/sex/dose). * *P* < 0.05 & ** *P* < 0.01 compared with normal group.

### Histopathogical findings

Histopathological examinations are an important aspect of safety assessments. In the histopathological examination, no noteworthy HZJW related lesions were observed, though some abnormalities were found. The major pathological findings from the histopathological examination included minimal inflammatory cell foci and vacuolar degeneration in the liver, portal myocardial inflammatory cell infiltration in the heart, peri-bronchovascular chronic inflammation and, inflammatory cell foci hyperplasia in the lungs, the tubule interstitial chronic inflammation, tubule cortical vacuolar degeneration and protein cast in the kidneys. However, these symptoms are part of chronic progressive nephropathy often observed in rats of old age and is particularly frequently observed in males. These findings were noted in both sexes in a manner not dependent on dose. The high-dose group (5000 mg/kg) did not differ significantly from the control group in any organ. These changes were not considered to be treatment related, because these microscopic changes were commonly observed in untreated old rats.

## Discussion

Currently, the commercial use of traditional herbal medicines has increased and various new drugs are being developed based on this development. Concerns have been raised over the lack of scientific evidence regarding the efficacy and safety of herbal products [23,24], though some have been verified by clinical trials. Many researchers have undertaken studies to validate the efficacy of herbal prescriptions; however, few have initiated investigations addressing their safety and toxicity. Indeed, the toxicity of many of these herbal remedies has not been scientifically validated, and their safeties have been questioned recently due to reports of side effects and fatalities [25], hepatotoxicity [26], and nephrotoxicity [27]. Considering the complexity of general herbal prescriptions and their inherent biological variation, it is necessary to evaluate their safety, efficacy, and quality [28]. In this study, we examined the efficacy and safety of HZJW, an herbal-derived anti-ulcer formulation applied in clinics, via gastroprotective assessment, anti-*H*. *pylori* assay, oral acute toxicity and a systemic 6-month repeated-dose toxicity study.

The gastroprotective effect of HZJW was investigated by two animal models of acute gastric injury induced by necrotizing agents, *i*.*e*., HCl/Ethanol and NSAIDs. The HCl/Ethanol method is a rapid and convenient way of screening agents of antiulcer potency, which is assessed in terms of absence or reduction in macroscopically visible lesions [29]. HCl/Ethanol acts by exerting a direct toxic effect on the epithelium, inducing the formation of characteristic necrotic lesions due to a reduction in the mucus. Besides, it causes reduction of gastric blood flow, solubilization of mucus and bicarbonate secretion [30]. HCl/Ethanol induced gastric damage ranging from endothelial microvascular damage to development of macroscopic gastric mucosal lesions, which can be mainly attributed to the inhibition of cytoprotective prostaglandins (PG) biosynthesis [31]. A number of mechanisms that include enhanced gastric mucosal defense through increasing mucus and/or bicarbonate production, reducing gastric acid secretion or by simply neutralizing the gastric acidity [32], can mediate the gastric mucosal protection against HCl/Ethanol. In the present study, the control group subjected to HCl/Ethanol clearly produced the expected characteristic zone of necrotizing mucosal lesions, while pretreatment with HZJW had significantly and dose-dependently decreased the ulcerative index and the percentage of lesion, thus markedly improved ulcer healing. These results indicated that HZJW exhibited protective effect against HCl/Ethanol-induced ulcerogenesis in rats.

Another experimental protocol employed in the investigation was NSAIDs- induced ulcer by aspirin, which induced gastric lesions due to the distinct mechanism in generating ulcer lesions compared with HCl/Ethanol. NSAIDs induce injury/bleeding via three key pathways: inhibition of cyclooxygenase (COX)-1 activity, inhibition of COX-2 activity, and direct cytotoxic effects on the epithelium [33]. The main importance of the systemic effects of NSAIDs, in terms of inducing gastric ulceration, is their ability to suppress prostaglandin synthesis [34]. In the stomach, prostaglandins play an important protective role by stimulating the secretion of bicarbonate and mucus, maintaining the blood flow of the mucosa, and they are responsible for regulating mucosal cell renewal [35]. Aspirin is a cyclooxygenase inhibitor which suppresses gastroduodenal bicarbonate secretion, reduces endogeneous prostaglandin biosynthesis and disrupts the mucosal barrier as well as mucosal blood flow in animals, causing increased susceptibility to gastric mucosal lesions [36]. In this ulcerogenesis model, the lesion index increased by aspirin was observed to be counteracted by pretreatment with HZJW of different dosages. The data obtained demonstrated that HZJW-treated groups displayed significant reduction in ulcerative lesion, as compared to their control counterparts. Based on the observations outlined, HZJW was in possession of good therapeutic action on the gastric ulcers. The protective effect of HZJW against the gastric damage might be due to possible encouragement of gastric mucosal defense and/or stimulation of endogeneous prostaglandins secretion. However, the precise mechanism underlying this specific action merited further exploration.

Eradication of *H*. *pylori* is an important objective in overcoming gastric diseases. *H*. *pylori* is considered the main etiological agent of human peptic ulcer, with a worldwide prevalence rate of about 40% in developed countries and over 80% in developing countries [37]. Indeed, half of all gastric ulcer cases are associated with infection by *H*. *pylori*[38,39]. Given the major role that *H*. *pylori* plays in the aetiology of peptic ulcers, its eradication is strongly recommended as key for the effective management of these pathologies [2]. In this investigation, the *in vitro* anti-*H*. *pylori* properties as well as *in vivo H*. *pylori* eradication activity of HZJW in infected mice was assessed. Rapid urease test (RUT), with its high sensitivity and specificity, is considered to be a reliable test for the initial diagnosis of *H*. *pylori* infection [40]. In the RUT assay, HZJW exhibited strong antagonistic activity against *H*. *pylori*. HZJW were able to significantly reduce the number of animals that presented a positive urease test, thus preventing the colonization of *H*. *pylori* in the stomach. The immunogold test and histopathological analysis further confirmed the *in vivo* anti-*H*. *pylori* activity of HZJW, in which HZJW could reduce the microorganism detection in histological sections, and thereby clearing *H*. *pylori* from the stomach of infected mice. All these results suggested the possible *in vivo* efficacy in eradication therapy for *H*. *pylori*, and this anti-*H*. *pylori* activity should contribute to the therapeutic effect of HZJW in treating gastrointestinal disorders.

A number of studies have shown that *H*. *pylori* eradication by antibiotic drugs (*i*.*e*., metronidazole, amoxicillin, and clarithromycin) is achieved by both systemic and topical action [41,42]. However, HZJW displayed stronger *in vivo* anti-*H*. *pylori* activity while weaker activity against *H*. *pylori in vitro*. This suggests that the anti-*H*. *pylori* capacity of HZJW might not result from the topical action but may take place by a systemic action that is attributed to the metabolic transformation of multi components in HZJW into active intermediates. It is known that clearance of *H*. *pylori* from the stomach of infected patients can be due to the direct topical activity of the ingested drugs at the gastric mucosal epithelium, and specially the secondary systemic therapeutic activity, which result from the back secretion and re-entry of the absorbed active principle from the basal to the apical side of the gastric epithelium. After absorption into the bloodstream, HZJW is postulated to be secreted, metabolized into active forms in the liver or during its trans-intestinal passage, from the basal side back to the apical surface of the gastric epithelium where *H*. *pylori* is located, thus improving the level of clinical treatment. However, further in-depth exploration was warranted.

The equilibrium between the therapeutic versus toxicological effects of a drug is a vital parameter in assessing its applicability in relation to pharmacological action [43]. As a part of this pharmacological study, HZJW was investigated for the acute and general toxicity in rodents. The data obtained in acute toxicity test by a procedure of fixed dose suggest that the oral LD_50_ of HZJW in mice was over 18.0 g/kg. Anatomical results presented the absence of abnormal organic damages in the dead mice. During the 6-month repeated-dose (1000, 2500, or 5000 mg/kg/day of HZJW) study, no drug-induced variations in clinical signs or in the ophthalmological, histopathological, hematological and blood biochemistry were observed in any of the test-article-treated groups compared to the recovery and control group. Throughout the experimental period, there were no significant changes noted in general behavior, skin effects, defecation and postural abnormalities in all animals. Although clinical indications like fur loss were observed in some rats, these symptoms generally occurred spontaneously in toxicity test due to systemic administration [44]. Additionally, these symptoms were infrequent and not dose-dependent. Therefore, this symptom was not considered to be a HZJW-induced abnormality. Some changes in other clinical observations, hematology, serum biochemistry, gross findings and organ weights were considered to be not dose-dependent and treatment-related, incidental and within the range of normal biological variation [45]. In addition, no corresponding histopathological findings were observed in related organs. Therefore, these were not considered to be triggered by HZJW.

In the histopathological examination, some rats presented with anomalous pathologies but no significant differences were found in relation to treatment with HZJW. No consistent treatment-related histopathological abnormalities were found in rats of either sex. These observations are readily detected in clinically normal rats of the age and were considered spontaneous or incidental in nature. Therefore, these findings observed sporadically in both sexes of the treatment groups without a dose–response relationship were not considered to be changes triggered by the administration of HZJW, according to the comparison to the recovery and vehicle control group. Based on these results, the No Observed Adverse Effect Level (NOAEL) of HZJW was considered to be 5,000 mg/kg/day for both sexes, a dose equivalent to 50 times of normal human dose in clinical prescription. In view of the doses of the components consumed, HZJW was extrapolated to offer a wide margin of safety by oral route. However, since toxicity in animals and humans is genetically diverse and may respond differently, especially with respect to conditions of gastrointestinal disorders, additional toxicological assessment in other species, such as dogs, needs to be performed to evaluate the safety of HZJM, and careful observation should be also conducted in clinical practice.

## Conclusions

Taken together the present results, the efficacy and safety of HZJW in healing peptic ulcer and combating *H*. *pylori* were demonstrated. The findings outlined corroborated their conventional indications, and contributed to their antiulcer pharmacological validation, lending more credence to clinical applications for the traditional treatment of stomach complaints symptomatic of peptic ulcer disease (PUD). Therefore, HZJW might have the potential for further development as a safe and effective alternative/complementary to conventional medication in treating gastrointestinal (GI) disorders.

## Abbreviations

TCM: Traditional Chinese Medicine; RUT: Rapid urease test; NSAID: Nonsteroidal anti-inflammatory drug; PUD: Peptic ulcer disease; MIC: Minimum inhibitory concentration; NOAEL: No Observed Adverse Effect Level; RBC: Red blood cell; HGB: Hemoglobin; HCT: Hematocrit; MCV: Mean corpuscular volume; MCH: Mean corpuscular hemoglobin; MCHC: Mean corpuscular hemoglobin concentration; RDW: Red cell distribution width; PLT: Platelets; MPV: Mean platelet volume; PDW: Platelet distribution width; WBC: White blood cell counts; RET: Reticulocytes; NE: Neutrophils; LYM: Lymphocytes; MON: Monocytes; EOS: Eosinophils; BAS: Basophils; PT: Prothrombin time; AST: Aspartate aminotransferase activity; ALT: Alanine aminotransferase activity; ALP: Alkaline phosphatase activity; ALB: Albumin; TP: Total protein; GLU: Glucose; CHOL: Total cholesterol; CPK: Creatine phosphokinase activity; T-BIL: Total bilirubin; D-BIL: Direct bilirubin; CRE: Creatinine; TG: Triglycerides; Ure: Urea; Na+: Sodium ions; K+: Potassium ions; Cl-: Chlorideions.

## Competing interest

The authors declare that there are no conflicts of interest.

## Authors’ contributions

All authors participated in the acquisition of data and revision of the manuscript. All authors conceived of the study, determined the design, interpreted the data and drafted the manuscript. All authors read and gave final approval for the version submitted for publication.

## Pre-publication history

The pre-publication history for this paper can be accessed here:

http://www.biomedcentral.com/1472-6882/13/119/prepub

## Supplementary Material

Additional file 1: Figure S1 HPLC Chromatogram of HZJW and its components. HZJW and its main compounds were subjected to HPLC. The chromatograms were obtained at 335 nm. (A): HZJW without *Coptidis Rhizoma Scutellarin*; (B) HZJW without *Scutellariae Barabtae Herba*; (C) HZJW without *Cynanchi Paniculati Radix et Rhizoma*; (D) Standard mixture of three major compounds: (1) scutellarin (10.35min), (2) berberine (21.67 min), (3) paeonol (25.94 min);(E) HZJW. Click here for file
