# Supplementary material for: Gastroprotective and anti-Helicobacter pylori potential of herbal formula HZJW: safety and efficacy assessment
Source: BMC Complement Altern Med. 2013 May 30;13:119. doi: 10.1186/1472-6882-13-119 (PMC3679842; doi:10.1186/1472-6882-13-119)
Supplement: Additional file 1: Figure S1 — HPLC Chromatogram of HZJW and its components. HZJW and its main compounds were subjected to HPLC. The chromatograms were obtained at 335 nm. (A): HZJW without Coptidis Rhizoma Scutellarin; (B) HZJW without Scutellariae Barabtae Herba; (C) HZJW without Cynanchi Paniculati Radix et Rhizoma; (D) Standard mixture of three major compounds: (1) scutellarin (10.35min), (2) berberine (21.67 min), (3) paeonol (25.94 min);(E) HZJW. [file 1472-6882-13-119-S1.doc]

**Proﬁling the chemical contents of HZJW by HPLC**

To assure the quality thereby warranting the safety and effectiveness of HZJW, the chromatographic profile of HZJW was established and characterized by high-performance liquid chromatography (HPLC) as described previously [11]. The chemicals used for the identiﬁcation and quantiﬁcation of compounds in HZJW included the following: scutellarin, [berberine](app:ds:berberine) and [paeonol](app:ds:paeonol), which originated from Scutellariae Barabtae Herba, Coptidis Rhizoma, and Cynanchi Paniculati Radix et Rhizoma, respectively. An ethanol standard stock solution containing the three typical chemicals was prepared and diluted to the appropriate concentration range, for the establishment of calibration curves. HZJW (500 mg) was dissolved in 25 mL of ethanol and ﬁltered through 0.45 μm syringe ﬁlter before injection into the HPLC system.

The Shimadzu LC solution-20A HPLC system (Shimadzu Co., Kyoto, Japan) consisted of a solvent-delivery unit, an online degasser, a column oven, an autosampler, and a photodiode array (PDA) detector. The analytical column used was Agilent Zorbax SB-C_18_ (250 mm×4.6 mm, particle size 5μm). The mobile phase consisted of acetonitrile (as Solvent A) and 20mmoL/L ammonium acetate (as Solvent B). The flow rate was set at 1.0 mL/min and the column temperature was maintained at room temperature. The pH of mobile phase was adjusted with [glacial](app:ds:glacial) [acetic](app:ds:acetic) [acid](app:ds:acid) to 4.0 ± 0.2. The linear gradient elution was applied from 15 to 25 % A from 0 to 10 min, from 25 to 35 % A from 10 to 12 min, from 35 to 50 % A from 12 to 25 min, from 50 to 15 % A from 25 to 30 min and at last 15 % A from 30 to 35 min. Samples of 10μL filtered through a 0.45 μm Millipore syringe were injected for HPLC analysis. Signals were detected at a wavelength of 335 nm. (Figure1S)

**(A)**

**(B)**

**(C)**

**(D)**


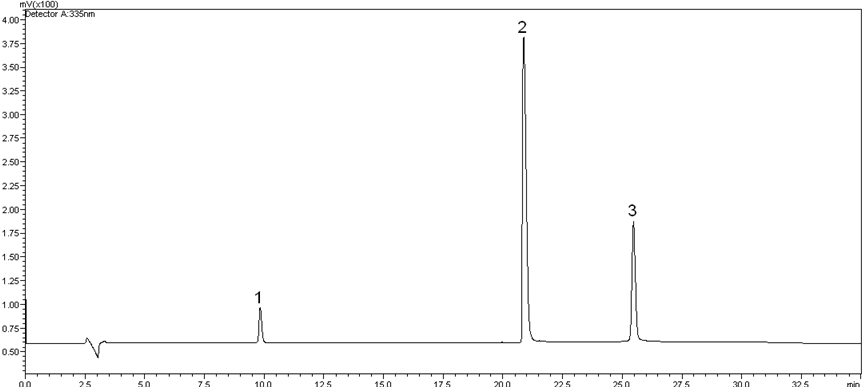


**(E)**


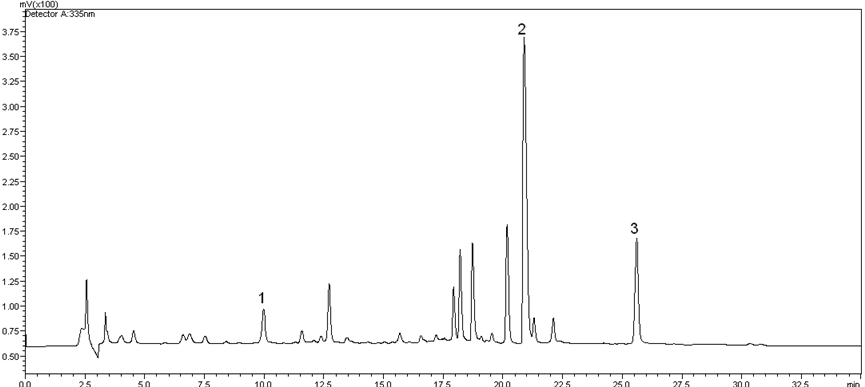


**Figure 1S** **Chromatogram of HZJW and its components**. HZJW and its main compounds were subjected to HPLC. The chromatograms were obtained at 335 nm. (A): HZJW without Coptidis Rhizoma Scutellarin; (B) HZJW without Scutellariae Barabtae Herba; (C) HZJW without Cynanchi Paniculati Radix et Rhizoma; (D) Standard mixture of three major compounds: (1) scutellarin (10.35min), (2) berberine (21.67 min), (3) paeonol (25.94 min)；(E) HZJW.
